# Genome-wide CRISPR screening identifies PHF8 as an effective therapeutic target for KRAS- or BRAF-mutant colorectal cancers

**DOI:** 10.1186/s13046-025-03338-2

**Published:** 2025-02-25

**Authors:** Zhao Liu, Yiqi Li, Simeng Wang, Yubo Wang, Mengjun Sui, Jiaxin Liu, Pu Chen, Jianling Wang, Yuchen Zhang, Chengxue Dang, Peng Hou

**Affiliations:** 1 Department of Endocrinology and International Joint Research Center for Tumor Precision Medicine of Shaanxi Province, The First Affiliated Hospital of Xi’an Jiaotong University, Xi’an, 710061 P.R. China; 2 Department of Surgical Oncology, The First Affiliated Hospital of Xi’an Jiaotong University, Xi’an, 710061 P.R. China; 3 Department of General Practice, Sir Run Run Shaw Hospital, Zhejiang University School of Medicine, Hangzhou, 310016 P.R. China; 4 Department of Vascular Surgery, The First Affiliated Hospital of Xi’an Jiaotong University, Xi’an, 710061 P.R. China; 5 Department of Nuclear Medicine, The First Affiliated Hospital of Xi’an Jiaotong University, Xi’an, 710061 P.R. China

**Keywords:** Colorectal cancer, PHF8, Immune escape, *KRAS* mutations, *BRAF* mutations

## Abstract

**Background:**

Mutations in *KRAS* and *BRAF* genes are prevalent in colorectal cancer (CRC), which strikingly promote tumorigenesis and lead to poor response to a variety of treatments including immunotherapy by activating the MAPK/ERK pathway. Thus, there is an urgent need to discover effective therapeutic targets and strategies.

**Methods:**

CRISPR-Cas9 lentiviral knockout library was used to screen the suppressors of anti-PD1 immunotherapy. Bioinformatic analysis was used to analyze the correlation between PHF8 expression and immune indicators in CRC. In vitro and in vivo experiments were utilized to determine the effects of PHF8 on the immune indexes and malignant phenotypes of CRC cells. qRT-PCR, western blotting, immunohistochemical (IHC) staining, and chromatin immunoprecipitation (ChIP)-qPCR assays were used to determine the regulatory effects of PHF8 on PD-L1, KRAS, BRAF, and c-Myc and the regulatory effect c-Myc/miR-22-3p signaling axis on PHF8 expression in CRC cells.

**Results:**

This study identified histone lysine demethylase PHF8 as a negative regulator for the efficacy of anti-PD1 therapy and found that it was highly expressed in CRCs and strongly associated with poor patient survival. Functional studies showed that PHF8 played an oncogenic role in KRAS- or BRAF-mutant CRC cells, but not in wild-type ones. Mechanistically, PHF8 up-regulated the expression of PD-L1, KRAS, BRAF, and c-Myc by increasing the levels of transcriptional activation marks H3K4me3 and H3K27ac and decreasing the levels of transcriptional repression mark H3K9me2 within their promoter regions, promoting immune escape and tumor progression. Besides, our data also demonstrated that PHF8 was up-regulated by the c-Myc/miR-22-3p signaling axis to form a positive feedback loop. Targeting PHF8 substantially improved the efficacy of anti-PD1 therapy and inhibited the malignant phenotypes of KRAS- or BRAF-mutant CRC cells.

**Conclusion:**

Our data demonstrate that PHF8 may be an effective therapeutic target for KRAS- or BRAF-mutant CRCs.

**Supplementary Information:**

The online version contains supplementary material available at 10.1186/s13046-025-03338-2.

## Introduction

It is commonly acknowledged that colorectal cancer (CRC) is a diverse disease, including a multitude of gene mutations and the activation of oncogenic pathways in its etiology [1], of which the most significant activating *KRAS* mutations occur in about 40% of cases, while *BRAF* mutations are observed in approximately 10% [2]. Patients with *KRAS* or *BRAF* mutations are associated with worse survival and show poor response to a variety of treatments such as radiotherapy, chemotherapy, targeted therapies, and immunotherapy compared with wild-type patients [2–10].

As a component of several growth factor signaling pathways, KRAS activation causes constitutive activation of the MAPK/ERK pathway, promoting cell proliferation, survival, differentiation, and metastasis [11]. B-Raf, a downstream effector of KRAS protein, is responsible for the activation of MEK1 and MEK2, causing the phosphorylation of ERK1 and ERK2 and the subsequent phosphorylation of enzymes that drive cell cycle progression [12]. In the progression of CRC, somatic *KRAS* and *BRAF* mutations lead to continued activation of the MAPK/ERK pathway and mediate multiple adverse biological effects [8]. For example, the presence of mutant KRAS is correlated with resistance to EGFR targeting therapies [13] and decreased lymphocyte infiltration in TCGA and KFSYSCC CRC datasets [10]. *KRAS* mutations also lead to the downregulation of MHC Class I molecules, impairing the ability of CD8 + cytotoxic T cells to detect cancer cells [14]. *BRAF* mutations drive constitutive activation of the MAPK/ERK pathway independently of RAS activity and promote malignant biological activities of cancer cells [15]. MAPK/ERK pathway has been demonstrated to be related to elevated PD-L1 expression and restrains anti-tumor immunity [16].

Mutant KRAS has long been considered an undruggable target due to the absence of hydrophobic pockets for drug binding, but efforts to inhibit KRAS activity are constantly evolving [17, 18]. Recently, inhibitors against the mutation of KRAS G12C, such as sotorasib (AMG-510) and adagrasib (MRTX849), have entered the clinical application stage [19]. However, the initial objective response rate (ORR) of sotorasib in KRAS G12C-mutant CRC patients was only 7% (3 out of 42 patients) [20], while the preliminary response rate of adagrasib was 17% (3 out of 18 patients) [21]. Disappointingly, all patients who received sotorasiba treatment and achieved initial objective remission eventually progressed. Moreover, the major challenge for metastatic CRC patients with *KRAS* mutations is that the prevalence of G12 hotspot mutations accounts for approximately 68%, while G12C is present in only 11% of cases [22]. BRAF inhibitors such as vemurafenib have been shown to be more effective in metastatic melanomas with *BRAF*^*V600E*^ mutation rather than CRCs because epidermal growth factor receptor (EGFR) on the surface of CRC cells can reactivate RAS and CRAF-mediated MAPK signaling pathway in the state of BRAF inhibition [23]. Thus, it remains crucial to identify new and efficient therapeutic targets for KRAS- or BRAF-mutant CRCs.

In this study, we identify histone lysine demethylase PHF8 as an immunotherapeutic target using CRISPR-Cas9 gene knockout library. PHF8 knockdown synergizes with anti-PD1 treatment to suppress tumor growth by down-regulating the levels of PD-L1. Also, we demonstrate that PHF8 plays an oncogenic role in KRAS- or BRAF-mutant CRC cells but not in wild-type ones by transcriptionally up-regulating the expression of *KRAS*, *BRAF,* and *c-Myc*. Therefore, PHF8 may be an effective therapeutic target for KRAS- or BRAF-mutant CRCs.

## Materials and methods

### Cell culture

Normal colon epithelial cell line NCM460 and human CRC cell lines HCT116, RKO, LOVO, SW480, and SW48 as well as murine colon adenocarcinoma cell lines MC38 and CT26.WT were obtained from the American Type Culture Collection and authenticated (ATCC; Manassas, VA, USA). At 37 °C, NCM460, HCT116, RKO, LOVO and CT26.WT cells were cultivated in RPMI 1640 medium containing 10% fetal bovine serum (FBS), while SW480, SW48, and MC38 cells were cultured in DMEM media containing 10% FBS. Using the One-Step Quickcolor Mycoplasma Detection Kit, mycoplasma was consistently eliminated from all cells (Shanghai Yise Medical Technology Co., Ltd).

### In vivo CRISPR-Cas9 screening in MC38 cell-derived allograft tumor model

The CRISPR-Cas9 lentiviral gene library used in this study was created by the team of Feng Zhang, and the virus was manufactured and packaged by Shanghai Genechem Co., LTD. The library contains 130,209 sgRNA sequences targeting 20,611 known functional genes (6 sgRNAs/gene). According to the manual provided by the company (http://www.genechem.com.cn) and the corresponding references [24, 25], we carried out the CRISPR-Cas9 screening. The mouse colon cancer cell line MC38 was then chosen as the tool cell for this investigation, and the MTT assay was used to determine the minimum inhibitory concentration of puromycin on MC38 cells. To construct allograft tumor models, we infected MC38 cells with a lower MOI and injected them into *14* C57BL/6 mice after puromycin screening. The mice models were then randomly separated into two groups: PBS control and PD1 antibody (BE0273, BioXCell). After two weeks, the mice were sacrificed and the genomic DNA of each tumor was collected and amplified using nested PCR with a fragment size of 299 bp. After gel electrophoresis and purification, the amplification products were sent to Genewiz (Suzhou, China) for amplicon sequencing. *Specific experimental process was shown in Figure*S1. The number of sgRNAs was determined by sequence extraction and filtering, and MAGeCK software was used to examine the decreased and enriched sgRNAs and their corresponding genes in the PD1 antibody-treated group. Finally, potential genes were identified by a series of stringent filtering.

### Reagents

PHF8 inhibitor daminozide was purchased from TargetMol (Boston, MA, USA) [26]. MEK1/2 inhibitor GSK1120212 (#S2673) and c-Myc inhibitor 10,074-G5 (#S8426) were purchased from Selleck Chemicals (Houston, TX, USA). All the reagents were used according to the manufacturer’s instructions.

### Human datasets and data analysis

The publicly available data section is provided in the expanded Materials and Methods section that is available in Supplementary data.

### Clinical samples

A total of 22 pairs of CRC tissues and adjacent non-cancerous colon tissues (control subjects) were obtained from 22 CRC patients who underwent surgery at The First Affiliated Hospital of Xi’an Jiaotong University. Before surgery, all patients did not receive any treatment interventions and signed an informed consent form. Two professional pathologists recognized the histological type of each tissue blindly and independently. Histopathological data of CRC patients were presented in Supplementary Table S1. This study was approved by the Institutional Review Board and Human Ethics Committee of the First Affiliated Hospital of Xi’an Jiaotong University.

### Immunohistochemistry (IHC) staining

The protocol was similarly performed according to a previous study [27]. The information on antibodies used in this study was presented in Supplementary Table S2.

### Lentivirus-mediated ectopic expression and knockdown of PHF8

The lentivirus encoding PHF8 and the lentivirus expressing shRNA targeting PHF8 (sh-PHF8#1 and sh-PHF8#2) as well as their control lentivirus were obtained from Shanghai Genechem Co., Ltd. A day before infection, cells were seeded and allowed to reach 30-50% confluence. Puromycin was used to select positive cells, which were then maintained in a solution containing a low dosage of puromycin for further tests. qRT-PCR and western blotting assays were used to validate the efficacy of overexpression or knockdown.

### siRNA-mediated knockdown of PHF8

siRNAs targeting PHF8 (si-PHF8#1 and si-PHF8#2) and control siRNA (si-NC) were purchased from RiboBio Co., Ltd. (Guangzhou, P. R. China). A final siRNA dose of 80 nmol/L was used to transfect cells with X-tremeGENE siRNA Transfection Reagent (Roche Diagnostics GmbH, Mannheim, Germany). The siRNA sequences were presented in Supplementary Table S3.

### RNA extraction and quantitative RT-PCR (qRT-PCR)

RNA isolation and qRT-PCR were performed according to a previous study [28]. *β-actin* was used to normalize the expression of target genes. The primer sequences were presented in Supplemental Table S4.

### Western blotting analysis

The detailed process was executed as previously mentioned [28]. The information of antibodies used in this study was provided in Supplementary Table S2.

### Animal studies

To construct allograft tumor models, a total of 5 × 10^5^ PHF8-knockdown MC38 cells and control cells were implanted into the inguinal region of 6- to 8-week-old female C57BL/6 mice. When tumor volumes reached 100 mm^3^ on day 8, these mice were randomly divided into four groups and received different treatments (Figure S2). Briefly, PD1 antibody (100 µg/mouse) was administered intraperitoneally four times every 3 days, while 100 mg/kg of daminozide was delivered intraperitoneally to each mouse for 10 consecutive days. Tumor volumes were measured every 2 days. *On the 20th day*, the blood samples were collected from the eyeballs of each mouse, mice were sacrificed and all tumors were collected and weighed. Tumor volumes were calculated by the following formula: tumor volume = length × width^2^ × 0.5. This study was approved by the Animal Ethics Committee of Xi’ an Jiaotong University.

### Flow cytometry analysis

The allograft tumor tissues were excised, chopped into pieces, and then incubated for 60 min at 37 °C in fresh medium containing 5 mg/mL collagenase and 1 U/mL DNase I. Single-cell suspensions were obtained by passing the homogenates through a 70 μm nylon mesh after washing with PBS. For examination of infiltrating CD4 + or CD8 + T lymphocytes in tumor tissues, cells were stained with APC fluorescence-labeled anti-CD3 (Cat. #100235, Biolegend), PE-labeled anti-CD45 (Cat. #103105, Biolegend), FITC-labeled anti-CD4 (Cat. #100405, Biolegend) and FITC-labeled anti-CD8 (Cat. #100705, Biolegend). All antibodies were diluted per the instructions and incubated with cells at room temperature for 30 min. Cell apoptosis was assessed using a flow cytometer (BD Biosciences), and the data were then analyzed using FlowJo software.

### Enzyme‑linked immunosorbent assay (ELISA)

The levels of TNFα, Granzyme B, and IFN-γ in allograft tumor tissues were measured by using the Mouse ELISA Kit (Jianglai Biotechnology, Shanghai, China) according to the manufacturer’s instructions.

### In vivo toxicity evaluation

Heart, liver, lung, and kidney tissues from mice with different treatments were collected for H&E staining assay. In addition, the blood taken from the eyeballs of mice was collected in a separate tube, left at room temperature for 1 h, centrifuged at 3000 rpm for 20 min, and the supernatant was extracted. According to the instructions for usage, the levels of alanine aminotransferase (ALT), aspartate aminotransferase (AST), creatinine (CRE), and blood urea nitrogen (BUN) in mouse serum samples were measured. The kits were purchased from Nanjing Jiancheng Bioengineering Institute. Specific procedures were available on the website (http://www.njjcbio.com).

### Chromatin immunoprecipitation (ChIP) assay

Using cell signaling technology ChIP Kit, the ChIP assay was performed following the operating instructions (CST Biotechnology). About 1–2 × 10^7^ collected cells were cross-linked with formaldehyde at room temperature for 10 min, followed by 5 min of quenching with glycine (final concentration 0.125 M) at room temperature. Whole-cell lysates were sonicated with VCX-130 PB (Sonics & Materials, Inc., Newtown, CT, USA) to fragment the chromatin. 10% of total chromatin from each lysate was utilized as an input control, and the remaining 90% was treated overnight in ChIP Buffer with 5 g of the specified antibodies. Non-specific IgG was used as a control. The immunoprecipitated protein DNA complex was then incubated at 4 °C for 2 h with ChIP Grade Protein A/G Magnetic Beads. Chromatin was eluted with ChIP Elution Buffer, and proteins were removed with 200 mM NaCl and 200 g/mL proteinase K at 65 °C for 2 h. Purified DNA was then utilized as a basis for further study. The primer sequences for ChIP-qPCR assays were presented in Supplementary Table S5. Each test was performed in triplicate.

### In vitro functional studies

Cell proliferation, colony formation, cell cycle, cell apoptosis, cell migration, and cell invasion assays were performed as described in a previous study [28].

### In vivo metastasis and imaging

About 5 × 10^5^ cells/100 µL luciferase-expressing PHF8-overexpression SW480 or SW48 cells and their control cells were injected into female NCG mice aged 7–8 weeks (Gempharmatech Co., Ltd) via the tail vein to construct a metastatic tumor mouse model. After a period of observation, mice were prepared for imaging to investigate tumor metastasis in vivo by intraperitoneal injection of D-fluorescein potassium. The specific usage method is as follows: D-fluorescein potassium salt was dissolved in D-PBS at a concentration of 15 mg/mL before being filtered through a 0.22 μm filter. The mice were injected with a 10 µL/g body weight solution of fluorescein potassium salt, waited for 10 to 20 min for the fluorescence signal to attain a stable plateau, and then imaged.

### Downregulation of miR-22-3p by MiRNA inhibitor

A final 80 nmol/L miR-22-3p inhibitor and control (Guangzhou RiboBio, Co., Ltd.) were transfected into CRC cells using X-tremeGENE siRNA Transfection Reagent (Roche Diagnostics GmbH, Mannheim, Germany) according to manufacturer’s instructions. After 48 h of transfection, the subsequent experimentation was conducted.

### Statistical analysis

Student’s *t*-test and two-way ANOVA with Bonferroni post-test were utilized for data comparison. SPSS statistical package 18.0 (IBM Corp., NY, USA) was used for statistical significance analysis. The data were presented in mean ± standard deviation (SD). *P* values below 0.05 were regarded as statistically significant.

## Results

### In vivo identification of immunosuppressive genes using CRISPR-Cas9 lentivirus gene knockout library

**Fig. 1:**
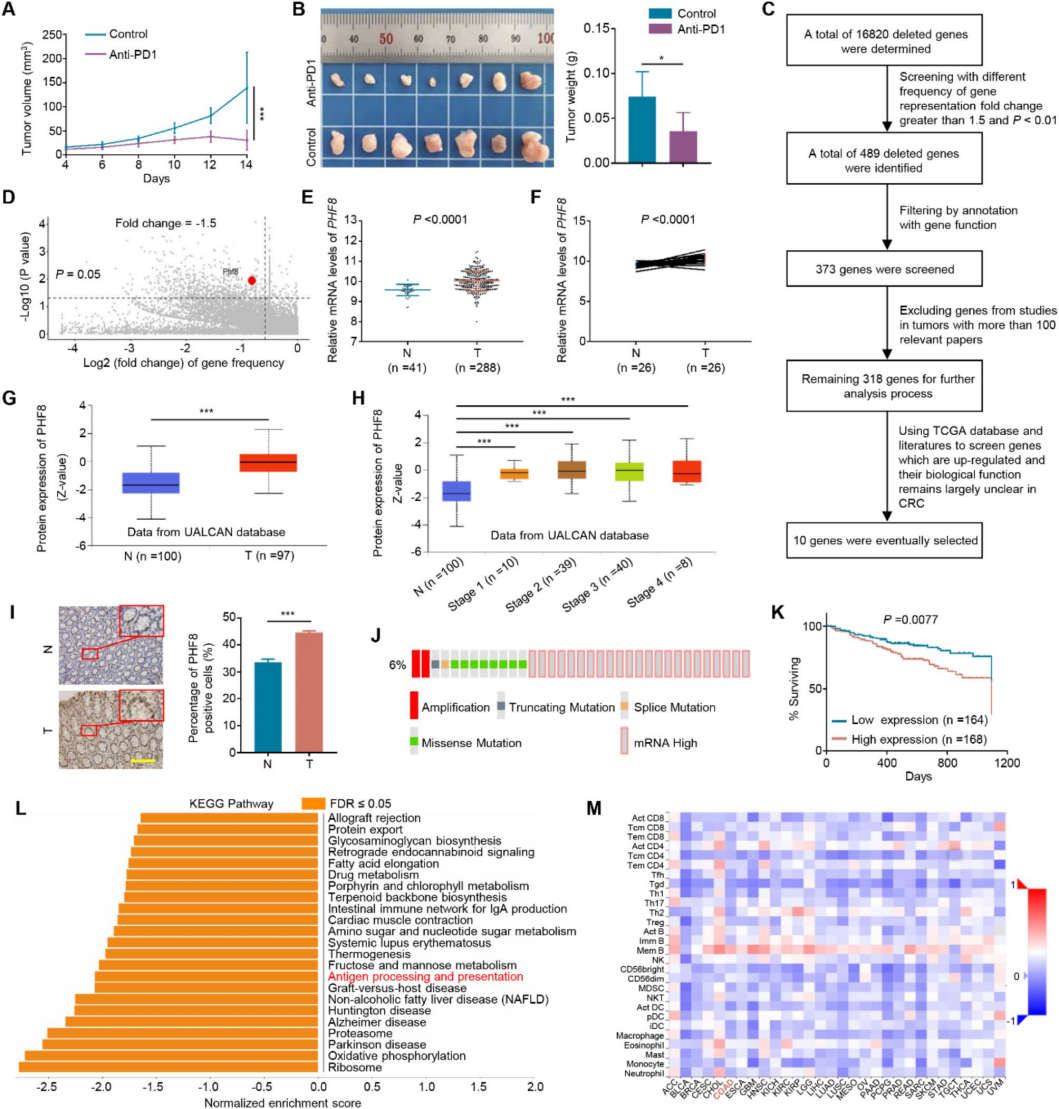
Identification of immunosuppressive genes via in vivo screen of CRISPR/Cas9 lentivirus gene knockout library. **A**. The growth curves of PD1 antibody-treated allograft tumors and control tumors in C57BL/6 mice (*n* = 7/group). **B**. Images of PD1 antibody-treated allograft tumors and control tumors (left panel) and statistical analysis of the weight of these tumors (right panel). **C**. Screening process of candidate genes. **D**. Distribution and identification of PHF8 in different frequencies of genes. **E**, **F**. Analysis of *PHF8* mRNA expression in CRCs (T) and non-cancerous colon tissues or their adjacent normal colon tissues (N) using TCGA database. **G**. Analysis of PHF8 protein expression in CRCs (T) and control subjects (N) by UALCAN data platform. **H**. Correlation analysis between PHF8 protein expression and clinicopathological stage using UALCAN data platform. **I**. Representative IHC staining of PHF8 (left panel) and the percentage of PHF8-positive cells (right panel) in CRCs (T) and control subjects (N). Scale bars, 200 μm. **J**. The alterations in the *PHF8* gene are found in a total of 6% (35/594) of CRC patients (data from cBioPortal). **K**. Correlation analysis between *PHF8* mRNA expression and the prognosis of CRC patients using the OncoLnc database. **L**. The pathways modulated by PHF8 were analyzed using the GSEA method. **M**. Correlation analysis between *PHF8* mRNA expression and infiltrating lymphocytes in different types of human cancers. COAD represents colon adenocarcinoma. *P* value in A was calculated using One-way analysis of variance (ANOVA). *P* values in B, E-H were calculated using two-tailed unpaired Student’s t-tests. *P* value in K was determined by the log-rank test. Data were presented as mean ± SD. *, *P* < 0.05; ***, *P* < 0.001

The CRISPR-Cas9 lentivirus gene knockout library used in this study contains 130,209 sgRNA sequences targeting 20,611 known functional genes (6 sgRNA/gene). The mouse colon cancer cell line MC38 was transduced with this lentivirus library, and the transduced cells were then expanded and injected subcutaneously into the groin of C57BL/6 mice to establish allograft tumor models. The mice were then randomly separated into groups receiving intraperitoneal injections of PBS (control group) and PD1 monoclonal antibody. The results showed that tumor growth in the PD1 antibody-treated group was significantly slower than that in the control group (Fig. 1A). Similarly, the tumor weight of the former was also lower than that of the latter (Fig. 1B).

The genomic DNA of each allograft tumor was then extracted, followed by nested PCR amplification of the sequences of sgRNAs. Next, MAGeCK software was used to examine the decreased and enriched sgRNAs as well as their respective genes in the PD1 antibody-treated group. The results demonstrated that the distribution of sgRNAs in the two groups was relatively uniform (Figure S3A), and the number of genes in the PD1-treated group and the control group was linearly symmetrical, indicating that *most genes did not affect the efficacy of anti-PD1 immunotherapy and only a few genes might be related to the outcome of anti-tumor immunity* (Figure S3B). A different frequency of gene representation in the MC38 allograft population derived from the anti-PD1 treated group was obtained when compared to the control group (Figure S4A; Supplementary data S1). GO and KEGG analysis showed that these deleted genes were not significantly enriched in biological processes (Figure S4B), molecular functions (Figure S4C), and signaling pathways (Figure S4D). However, we noticed that these genes were relatively abundant in TLR-related signaling pathways (Toll-like receptor) (Figure S4D).

According to the fluctuation in the number of sgRNAs, we established a series of stringent filtering parameters to screen 10 candidate genes (Fig. 1C; Supplementary data S2). Further analysis revealed a significant reduction in histone lysine demethylase PHF8 in the PD1 antibody-treated group (Fig. 1D), and there are studies demonstrating that PHF8 depletion induces a viral mimicry response in human colorectal adenocarcinoma by activating canonical immune signatures [29], supporting the selection of PHF8 for subsequent exploration. Using the UALCAN database, we discovered that *PHF8* expression in digestive tract cancers was significantly higher than that in adjacent non-cancerous tissues (Figure S5). Similarly, we found a significant elevation in mRNA expression (Fig. 1E-F) and protein expression (Fig. 1G) of PHF8 in CRCs compared to control subjects by analyzing the TCGA and UALCAN databases. However, there was no statistical difference in the expression levels of *PHF8* in patients with different clinical stages (Fig. 1H). We also performed immunohistochemical staining to assess PHF8 expression in a panel of CRC tissues and control subjects (Fig. 1I), further supporting the above conclusion. In addition, we found that *PHF8* was abnormally altered in about 6% of CRC tissues and was dominated by high mRNA expression using the cBioPortal database (Fig. 1J). Further analysis showed that high expression of *PHF8* was significantly related to poor patient survival (Fig. 1K). Univariate and multivariate prognostic analysis indicated that PHF8 might be a high-risk factor for the prognosis of CRC patients (Figure S6A-B), which was consistent with the nomogram result (Figure S6C). These findings imply that PHF8 may play a tumor-promoting role in CRC.

To determine whether PHF8 is involved in immune regulation, we used GESA enrichment analysis to discover that PHF8 could negatively modulate the antigen presentation process (Fig. 1L) and the response to interleukin-12 and the MHC protein complex (Figure S7A-B). The analysis of tumor-immune interaction further showed that the expression of *PHF8* in CRCs was inversely connected with multiple immune infiltrating lymphocytes (Fig. 1M), suggesting that PHF8 may hinder the immune response process of the tumor. Our data, taken together, suggest that PHF8 serves as an immunosuppressive factor for CRC.

### PHF8 negatively regulates antigen presentation, interferon response, and chemokine expression

**Fig. 2:**
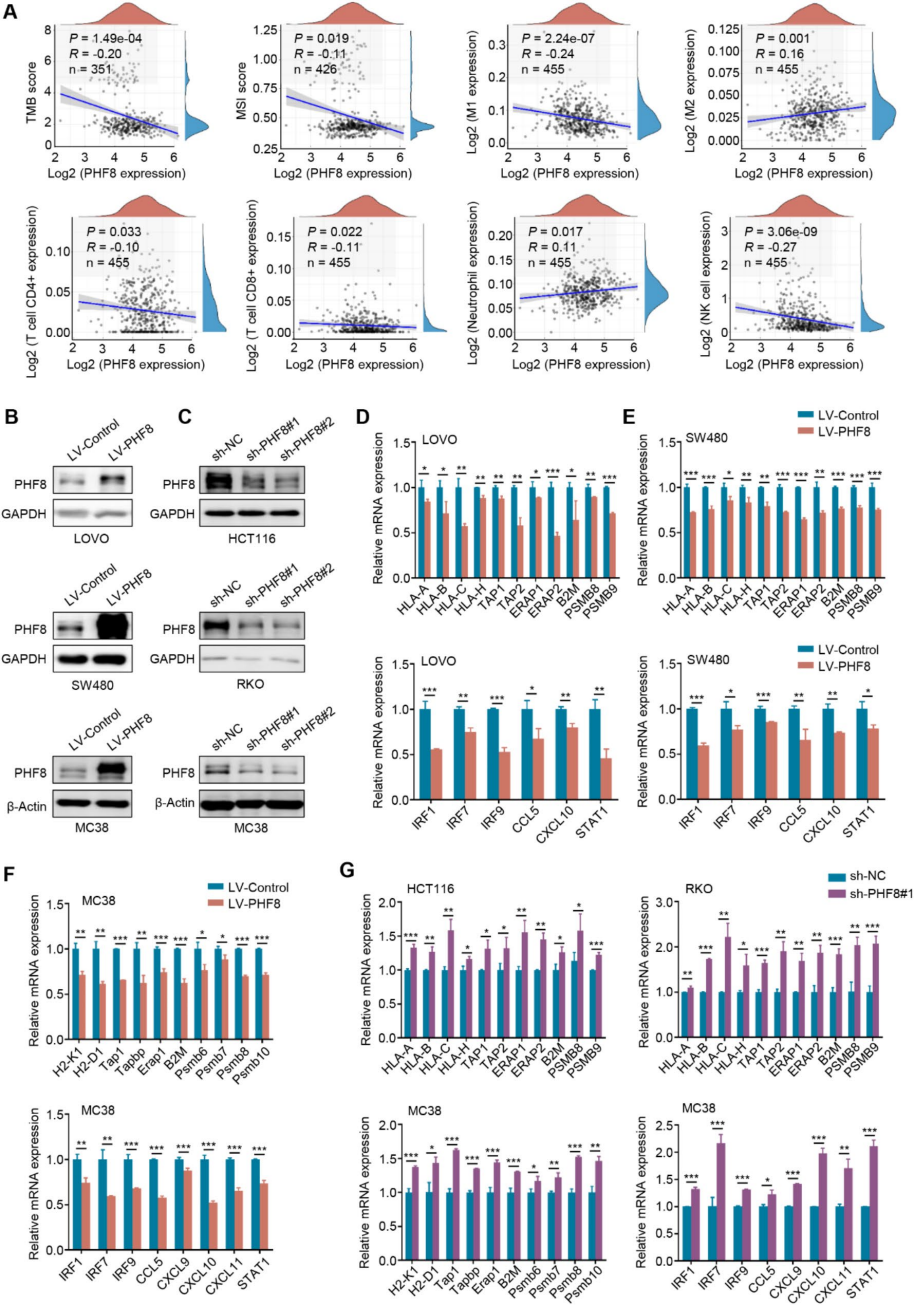
Negative regulation of antigen presentation, interferon response, and chemokine expression by PHF8. **A**. Correlation analysis of PHF8 with immune indexes in CRCs including tumor mutation burden (TMB), microsatellite instability (MSI), M1 macrophages, M2 macrophages, CD4 + T cells, CD8 + T cells, neutrophils, and NK cells. **B**, **C**. Ectopic expression of PHF8 in LOVO, SW480, and MC38 cells (**B**) and knockdown PHF8 in HCT116, RKO, and MC38 cells (**C**) were confirmed by western blotting analysis. GAPDH or β-actin was used as a loading control. **D-F**. The effects of ectopic expression of PHF8 in LOVO, SW480, and MC38 cells on the expression of MHC-I genes, interferon regulatory factors, response genes, and chemokines were determined by qRT-PCR assays. **G**. The effects of knocking down PHF8 in HCT116, RKO, and MC38 cells on the levels of related antigen-presenting genes, interferon regulatory factors, response genes, and chemokines were also determined by qRT-PCR assays. *β-actin* was used as the normalized control. *P* value in A was calculated by the log-rank test and the R-value was analyzed using Spearman’s correlation test. *P* values in D-G were calculated using two-tailed unpaired Student’s t-tests. Data were presented as mean ± SD. *, *P* < 0.05; **, *P* < 0.01; ***, *P* < 0.001

Further examining the immune-related factors associated with tumors, we found that PHF8 was negatively correlated with tumor mutation burden (TMB), microsatellite instability (MSI), M1 macrophages, CD4 + T cells, CD8 + T cells and NK cells, but positively correlated with M2 macrophages and neutrophils (Fig. 2A). In addition, the tumor-immune correlation analysis demonstrated that PHF8 expression was strongly negatively associated with the levels of MHC class I *genes* including B2M, HLA-A/B/C, TAP1/2 as well as chemokines such as CCL5 and CXCL10 (Figure S8A-B). Next, *according to the basal levels of PHF8 in different CRC cell lines* (Figure S9), we ectopically expressed or knocked down PHF8 in LOVO, SW480, HCT116, RKO, and MC38 cells (Fig. 2B-C), and determined their effect on the expression of the above immune-related genes. The results showed that ectopic expression of PHF8 in LOVO, SW480 and MC38 cells markedly downregulated the expression of MHC-I *genes*, interferon regulatory factors, response genes and chemokines (Fig. 2D-F), while knocking down PHF8 in these cells upregulated their expression (Fig. 2G). These results were similarly consistent with a previous study [29], suggesting that PHF8 inhibits immune response in tumors.

### Targeting PHF8 enhances the efficacy of PD1 antibody

**Fig. 3:**
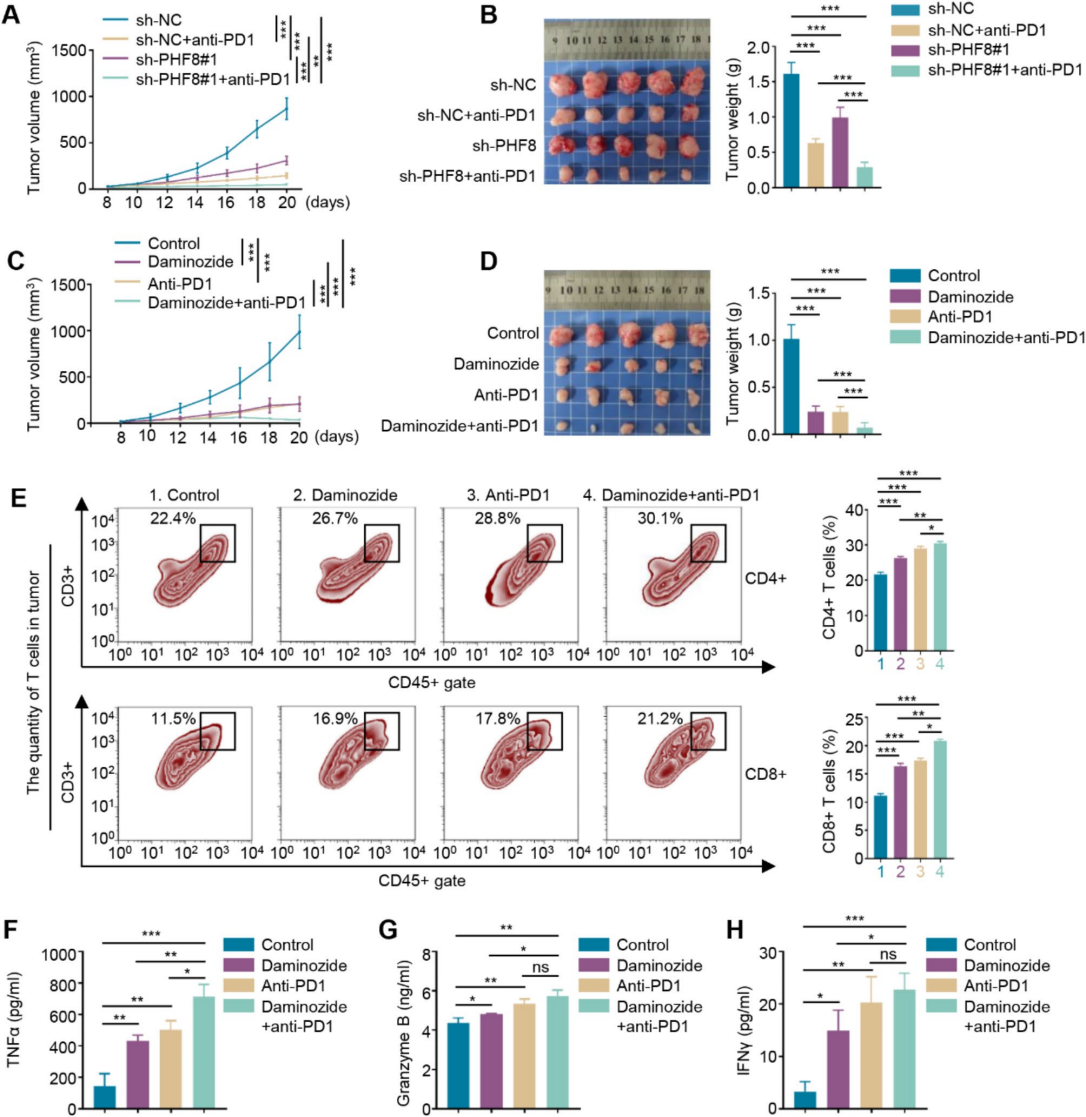
Inhibition of PHF8 improves the efficacy of PD1 antibody. **A**. The growth curves of PHF8-knockdown allograft tumors and control tumors with or without PD1 antibody treatment in C57BL/6 mice (*n* = 5/group). **B**. Images of the indicated allograft tumors (left panel) and statistical analysis of tumor weight (right panel). **C**. The growth curves of allograft tumors in C57BL/6 mice treated with PHF8 inhibitor daminozide and PD1 antibody, individually or in combination (*n* = 5/group). **D**. Images of the indicated tumors (left panel) and statistical analysis of tumor weight (right panel). **E**. Flow cytometry detecting the number of tumor infiltration CD4 +/CD8 + T cells in tumor tissues with the indicated treatments (left panels). Quantification analysis of cell numbers was shown on the right panels. **F-H**. ELISA assays were performed to measure the levels of TNFα (**F**), Granzyme B (**G**), and IFN-γ (**H**) in tumor tissues with the indicated treatments. *P* values in A and C were calculated by two-way ANOVA with Tukey’s multiple comparisons test. *P* values in B, D, and E-H were calculated using two-tailed unpaired Student’s t-tests. Data were presented as mean ± SD. *, *P* < 0.05; **, *P* < 0.01; ***, *P* < 0.001; ns, no significance

To verify the role of PHF8 in regulating the immune response, we established *allograft* tumor models by injecting PHF8-knockdown MC38 cells and control cells into the inguinal region of C57BL/6 mice and divided them into two groups, respectively. Two groups of mice were administrated with PD1 antibody every three days for 12 days (sh-NC + anti-PD1 and sh-PHF8#1 + anti-PD1), while the other two groups received PBS treatment (sh-NC and sh-PHF8#1). The results showed that both PHF8 knockdown and PD1 antibody slowed tumor growth and reduced tumor weight compared to the control, while PHF8 knockdown further enhanced the efficacy of PD1 antibody (Fig. 3A-B). To confirm the above findings, we established MC38 cell-derived *allograft* tumor models and administrated these mice with PHF8 inhibitor daminozide and PD1 antibody, individually or in combination. The results showed that tumors in daminozide- and PD1 antibody-treated groups grew much more slowly than the control group, while combination treatment was more effective than monotherapy (Fig. 3C). After 20 days of administration, the tumors from each group of mice were collected and weighted. The results further supported the above conclusion (Fig. 3D). In addition, we also performed IHC staining of Ki-67 in the above tumor tissues and demonstrated that the combination of PHF8 knockdown or daminozide and PD1 antibody greatly reduced the percentage of Ki-67 positive cells compared to monotherapy (Figure S10A-B).

We next explored the effect of the above treatments on anti-tumor immunity and found that, compared to monotherapy or control, daminozide combined with PD1 antibody significantly increased the infiltration of CD4 + and CD8 + T cells (Fig. 3E) and the levels of TNFα (Fig. 3F), Granzyme B (Fig. 3G) and IFN-γ (Fig. 3H) in tumor tissues. Importantly, we did not find obvious toxicity in mice with the above treatments, as reflected by no significant changes in the histology of major organs including heart, liver, lung, and kidney(Figure S11A). As support, we also failed to find significant changes in the levels of ALT, AST, CRE, and BUN among these mice with different administrations (Figure S11B-E), suggesting that these treatments did not cause severe hepatorenal toxicity. Collectively, PHF8 is a potential therapeutic target for CRC, while targeting PHF8 can improve the efficacy of PD1 immunotherapy with good biosafety.

### PHF8 elevates PD-L1 transcription via reprogramming of histone modifications within its promoter

**Fig. 4:**
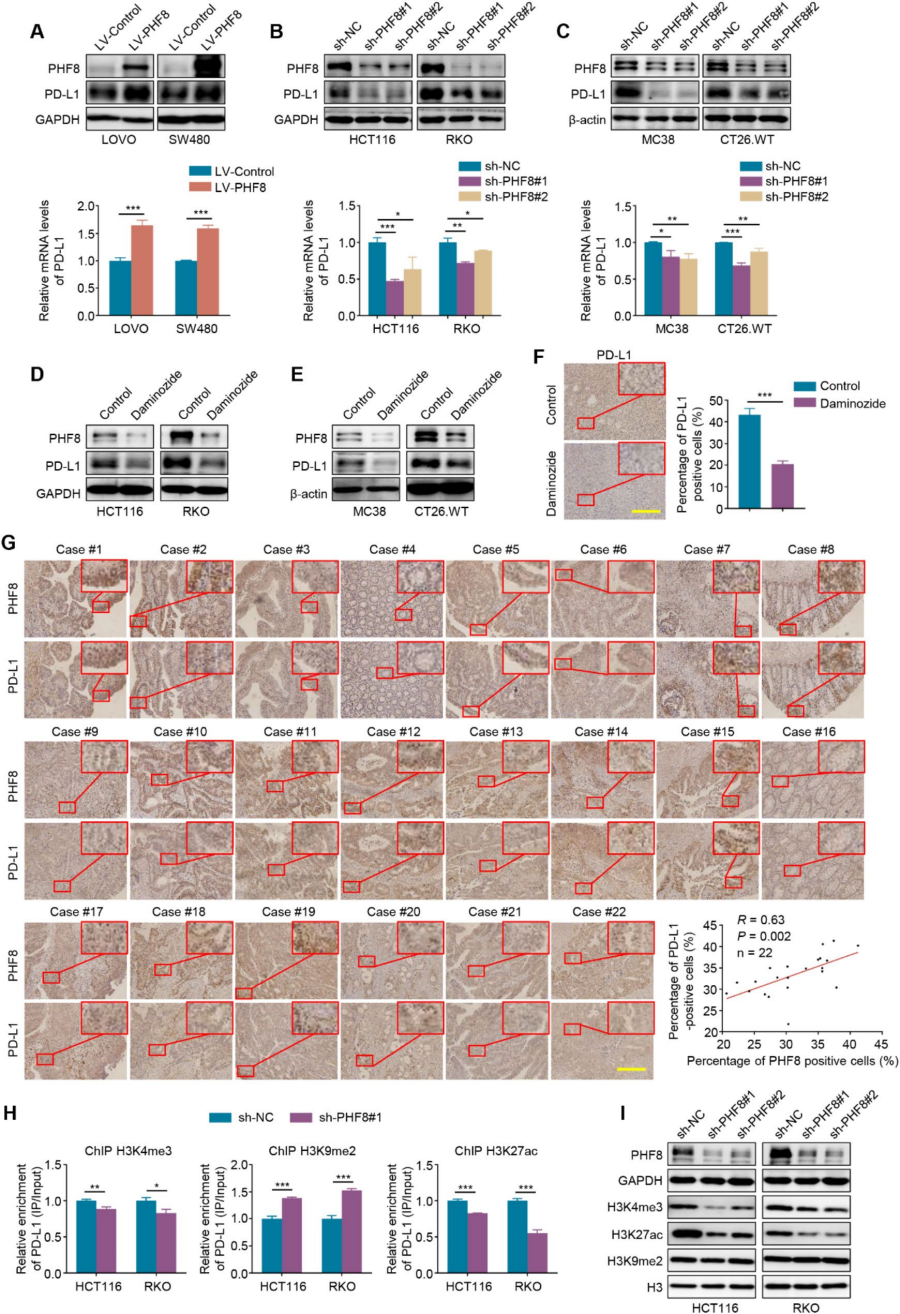
PHF8 enhances PD-L1 transcription by remodeling histone modifications within its promoter. **A**. Western blotting (upper panel) and qRT-PCR (lower panel) assays were used to determine the effects of ectopic expression of PHF8 in LOVO and SW480 cells on protein and mRNA expression of PD-L1. **B**. The effects of PHF8 knockdown on protein and mRNA expression of PD-L1 were determined by western blotting (upper panel) and qRT-PCR (lower panel) assays in HCT116 and RKO cells. **C**. Western blotting (upper panel) and qRT-PCR (lower panel) assays were performed to evaluate the effects of PHF8 knockdown on protein and mRNA expression of PD-L1 in MC38 and CT26.WT cells. **D**, **E**. HCT116 and RKO cells (**D**) as well as MC38 and CT26.WT cells (**E**) were treated with 50 µM PHF8 inhibitor daminozide for 48 h. Western blotting analysis was then used to evaluate its effect on the expression of PHF8 and PD-L1. **F**. The left panel shows the representative IHC staining of PD-L1 in daminozide-treated allograft tumors and control tumors, and the right panel shows the percentage of PD-L1-positive cells. Scale bars, 200 μm. **G**. IHC staining of PHF8 and PD-L1 in a total of 22 CRC tissues. Correlation analysis of PHF8 and PD-L1 protein expression was shown in the lower-right panel. **H**. ChIP-qPCR assays were performed in PHF8-knockdown HCT116 and RKO cells and their control cells to detect the levels of H3K4me3, H3K27ac, and H3K9me2 within the PD-L1 promoter. **I**. PHF8 was knocked down in HCT116 and RKO cells, and western blotting analysis was then used to evaluate its effect on the levels of H3K4me3, H3K27ac, and H3K9me2. GAPDH, β-actin, or H3 was used as a loading control. *P* value in G was calculated by the log-rank test and the R-value was analyzed using Spearman’s correlation test. *P* values in A-C, F, and H were calculated using two-tailed unpaired Student’s t-tests. Data were presented as mean ± SD. *, *P* < 0.05; **, *P* < 0.01; ***, *P* < 0.001

High expression of PD-L1 in tumor tissue has been shown to impair the immune response in vivo [30, 31], while PHF8 as a transcription coactivator is highly expressed in a variety of tumor types. Thus, we speculate that PHF8 may diminish the efficacy of immunotherapy by upregulating PD-L1 expression in CRC. Firstly, we ectopically expressed PHF8 in LOVO and SW480 cells, and discovered that ectopic expression of PHF8 significantly increased the protein and mRNA expression of PD-L1 (Fig. 4A). In contrast, knocking down PHF8 in HCT116 and RKO cells markedly decreased its protein and mRNA expression (Fig. 4B). Similar results were also observed in two murine-derived CRC cell lines MC38 and CT26 (Fig. 4C). This was supported by the results in PHF8 inhibitor daminozide-treated human- and murine-derived CRC cells (Fig. 4D-E) as well as daminozide-treated *allograft* tumor tissues (Fig. 4F). Meanwhile, we also performed IHC staining of PHF8 and PD-L1 in 22 primary CRC samples, and found a strong positive correlation between the expression levels of PHF8 and PD-L1 (Fig. 4G), further supporting the above conclusion.

Considering that PHF8 acts as a histone demethylase and transcription coactivator [32, 33], we thus suppose that PHF8 may remodel histone modifications within the PD-L1 promoter to alter its transcription activity. The results of ChIP assays indicated that knocking down PHF8 in HCT116 and RKO cells significantly decreased the levels of transcriptional activation marks H3K4me3 and H3K27ac within PD-L1 promoter while increasing the levels of transcriptional repression mark H3K9me2 within its promoter (Fig. 4H). This was also supported by the results of western blotting analysis (Fig. 4I). Our data, taken together, indicate that PHF8 upregulates PD-L1 expression in CRC by remodeling histone modifications within its promoter.

### PHF8 acts an oncogenic role in BRAF- or KRAS-mutant CRC cells but not in wild-type ones

**Fig. 5:**
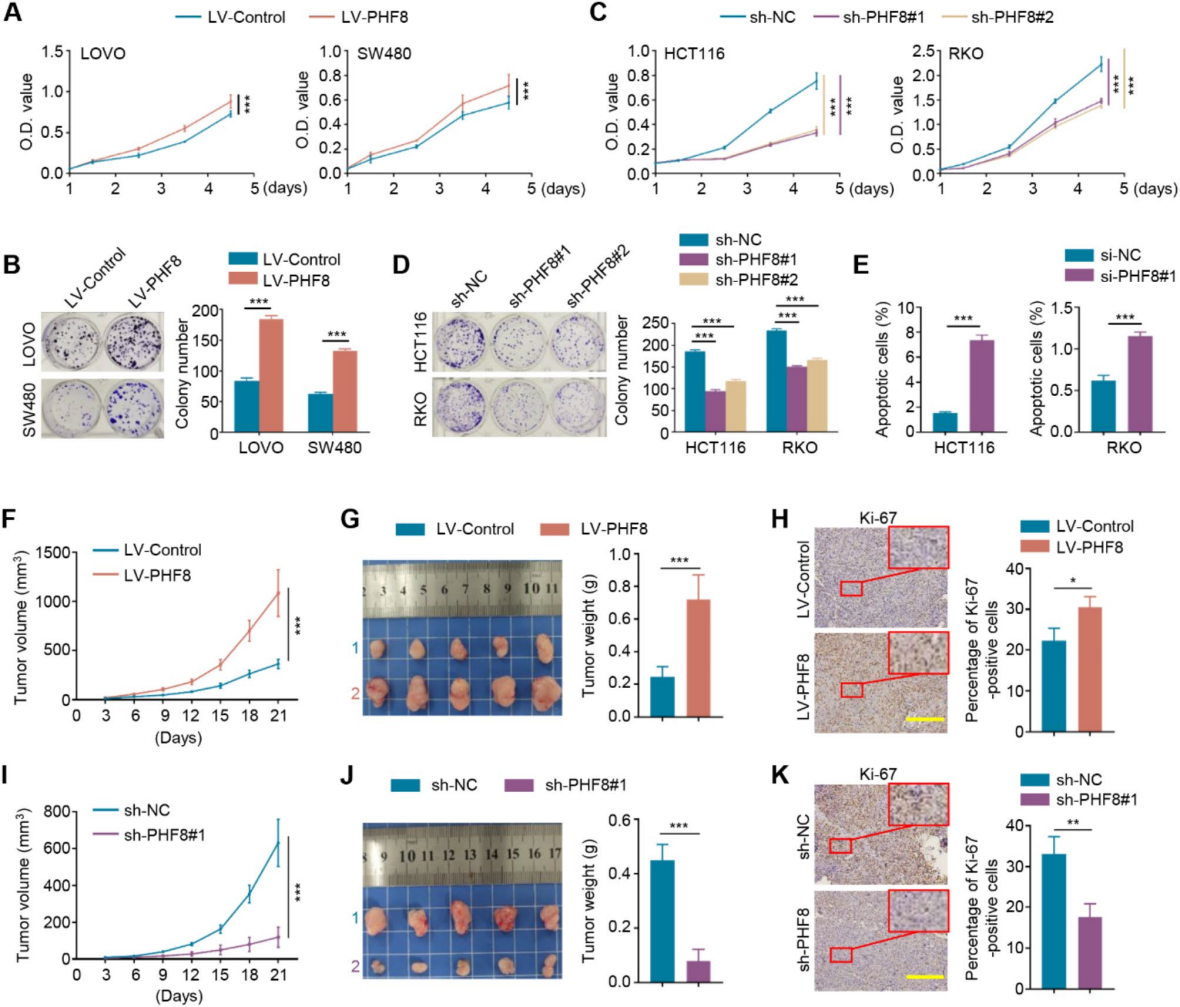
The oncogenic role of PHF8 in BRAF- or KRAS-mutant CRC cells. **A**. MTT assay showing the effect of PHF8 overexpression on the proliferation of LOVO and SW480 cells. **B**. The effect of PHF8 overexpression on colony formation ability of LOVO and SW480 cells. The left panel shows representative images of colony formation, and the right panel shows a quantitative analysis of colony numbers. **C**. MTT assays were performed to assess the effect of PHF8 knockdown on the proliferation of HCT116 and RKO cells. **D**. The effect of PHF8 knockdown on colony formation ability of HCT116 and RKO cells. Representative images of colony formation were shown in the left panel, and quantitative analysis of colony numbers was shown in the right panel. **E**. The effect of PHF8 knockdown on the apoptosis of HCT116 and RKO cells was determined by flow cytometry. **F**. The growth curves of PHF8-overexpression LOVO cell-derived xenograft tumors and control tumors in nude mice (*n* = 5/group). **G**. Images of the indicated xenograft tumors (left panel) and statistical analysis of tumor weight (right panel). **H**. Left panel shows the representative IHC staining of Ki-67 in the indicated xenograft tumors. Scale bars, 200 μm., The right panel shows a statistical analysis of the percentage of Ki-67-positive cells. **I**. The growth curves of PHF8-knockdown HCT116 cell-derived xenograft tumors and control tumors in nude mice (*n* = 5/group). **J**. Images of the indicated xenograft tumors (left panel) and statistical analysis of tumor weight (right panel). **K**. The representative IHC staining of Ki-67 in the indicated xenograft tumors was shown in the left panel, and statistical analysis on the percentage of Ki-67-positive cells was shown in the right panel. Scale bars, 200 μm. *P* values in A, C, F and I were calculated using One-way analysis of variance (ANOVA). *P* values in B, D, E, G, H, J, and K were calculated using two-tailed unpaired Student’s t-tests. Data were presented asmean ± SD. *, *P* < 0.05; **, *P* < 0.01; ***, *P* < 0.001

Given that PHF8 is highly expressed in CRCs and correlates with poor patient prognosis, we next explore its biological role in CRC cells. Firstly, we ectopically expressed PHF8 in LOVO and SW480 cells and found that PHF8 overexpression significantly enhanced their cell proliferation (Fig. 5A) and clone formation (Fig. 5B) compared to the control. *The same trend was also observed in HCT116 and RKO cells* (Figure S12A-C). In contrast, knocking down PHF8 in HCT116 and RKO cells suppressed their cell proliferation (Fig. 5C) and colony formation (Fig. 5D) compared to the control, *which was also supported by the data in LOVO and SW480 cells* (Figure S12D-F). As expected, we observed that PHF8 knockdown markedly induced the apoptosis of HCT116 and RKO cells (Fig. 5E). We next established xenograft tumor models by subcutaneously injecting PHF8-overexpressing LOVO cells or PHF8-knockdown HCT116 cells and their control cells into the groin skin of nude mice. The results showed that ectopic expression of PHF8 dramatically accelerated tumor growth (Fig. 5F) and increased tumor weight (Fig. 5G) compared to the control. Expectedly, the proportion of Ki-67-positive cells was higher in PHF8-overexpressing tumors than in control tumors (Fig. 5H). In contrast, PHF8 knockdown significantly delayed tumor growth (Fig. 5I) and reduced tumor weight (Fig. 5J) and the proportion of Ki-67-positive cells (Fig. 5K). These results suggest that PHF8 functions as an oncogene in CRC cells, which was consistent with a previous study showing that knocking down PHF8 in HCT116 cells significantly inhibited cell growth in vitro and *vivo* [34]. Surprisingly, when PHF8 was ectopically expressed in SW48 cells or Phf8 was knocked down in two murine-derived CRC cell lines MC38 and CT26.WT, we failed to find their significant effect on cell proliferation (Figure S13). The question is what reasons cause this contradictory conclusion? By investigating the genetic background of these CRC cell lines, we found that LOVO, SW480, and HCT116 cells carry mutant KRAS, RKO cells carry mutant BRAF, while SW48, MC38, and CT26.WT cells carry wild-type KRAS and BRAF. Based on these observations, we conclude that PHF8 acts an oncogenic role in KRAS- or BRAF-mutant CRC cells but not in wild-type ones.

**Fig. 6:**
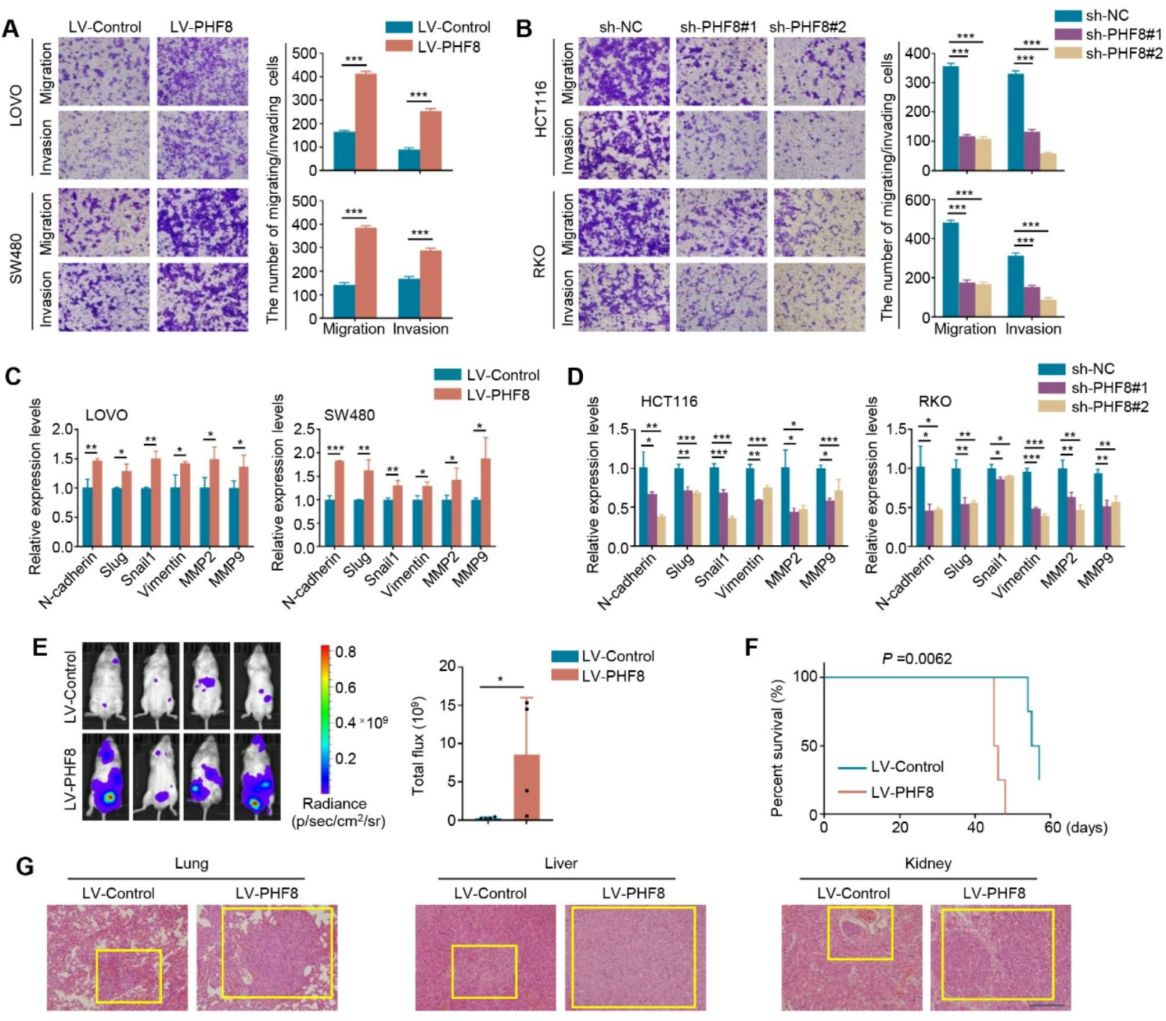
PHF8 enhances the metastatic ability of BRAF- or KRAS-mutant CRC cells. **A**. The effect of PHF8 overexpression on the migration and invasion potential of LOVO and SW480 cells. Representative images of migrated/invaded cells were shown in the left panel, and statistical analysis on the number of migrated/invaded cells was shown in the right panel. **B**. The effect of PHF8 knockdown on the migration and invasion potential of HCT116 and RKO cells. The left panel shows the representative images of migrated/invaded cells, and the right panel shows statistical data on the number of migrated/invaded cells. **C**. qRT-PCR assays were performed to assess the effect of ectopic expression of PHF8 on mRNA expression of N-cadherin, Slug, Snail Vimentin, MMP2, and MMP9 in LOVO and SW480 cells. **D**. The effect of PHF8 knockdown on mRNA expression of the above metastasis-related genes was determined by qRT-PCR assays in HCT116 and RKO cells. *β-actin* was used as the normalized control. **E**. The systemic metastasis of mice after tail vein injection of PHF8-overexpression SW480 cells and control cells was evaluated by an in vivo imaging system (left panel), and statistical results of bioluminescence in mice were shown on the right panel. **F**. The effect of tail vein injection of PHF8-overexpression SW480 cells and control cells on the survival of mice. **G**. Representative H&E images of metastatic tumors in lung, liver, and kidney tissues of mice after tail vein injection of PHF8-overexpression SW480 cells and control cells. The yellow rectangles were used to mark the location of the tumor. *P* values in A-E were calculated using two-tailed unpaired Student’s t-tests. *P* value in F was determined by the log-rank test. Data were presented as mean ± SD. *, *P* < 0.05; **, *P* < 0.01; ***, *P* < 0.001

We also evaluated the effect of ectopic expression or knockdown of PHF8 on the migration and invasion of CRC cells and demonstrated that ectopic expression of PHF8 in LOVO and SW480 cells significantly promoted cell invasiveness (Fig. 6A), while knocking down PHF8 in HCT116 and RKO cells suppressed their invasiveness ability (Fig. 6B). The similar results were also observed in HCT116 and RKO cells upon PHF8 overexpression and LOVO and SW480 cells upon PHF8 knockdown (Figure S14). Given that epithelial-mesenchymal transition (EMT) and matrix metalloproteinases (MMPs) play crucial roles in the process of tumor metastasis [35, 36], we next examined the effect of PHF8 overexpression or knockdown on the expression of several EMT-related genes and MMPs. The results indicated that, compared to the control, ectopic expression of PHF8 increased the mRNA levels of N-cadherin, Slug, Snail1, Vimentin, MMP9, and MMP2 in LOVO and SW480 cells (Fig. 6C), while knockdown of PHF8 in HCT116 and RKO cells dramatically reduced their mRNA and protein levels (Fig. 6D; Figure S15).

To confirm their conclusion, we constructed a metastatic tumor mouse model by injecting luciferase-expressing PHF8-overexpression SW480 cells and control cells into NCG mice via the tail vein. Bioluminescence imaging was then used to evaluate tumor metastasis. The results showed that PHF8 overexpression substantially increased the systemic metastasis ability of SW480 cells compared to the control (Fig. 6E). To be consistent with this, a significantly shorter survival time was observed in mice injected with PHF8-overexpression SW480 cells than control mice (Fig. 6F). In addition, we performed H&E staining of the lung, liver and kidney tissues from mice injected with PHF8-overexpressing SW480 cells and control cells, and found that PHF8 overexpression led to a considerably larger metastatic focus compared to the control (Fig. 6G). Similar experiments were also performed in BRAF or KRAS wild-type CRC cell line SW48. We constructed a metastatic tumor mouse model by injecting luciferase-expressing PHF8-overexpressing SW48 cells and control cells into NCG mice via the tail vein. Unfortunately, we failed to observe obvious metastasis in either the control group or the PHF8-overexpression group (Figure S16). This conclusion was also supported by the Transwell assays (data not shown). The possible reason is that SW48 cells have poor metastatic ability due to their BRAF or KRAS wild-type genetic background. These findings further support the oncogenic role of PHF8 in KRAS-mutant CRC cells.

### PHF8 activates the MAPK/ERK/c-Myc signaling pathway by remodeling histone modifications within the promoters of *KRAS*, *BRAF,* and *c-Myc*

**Fig. 7:**
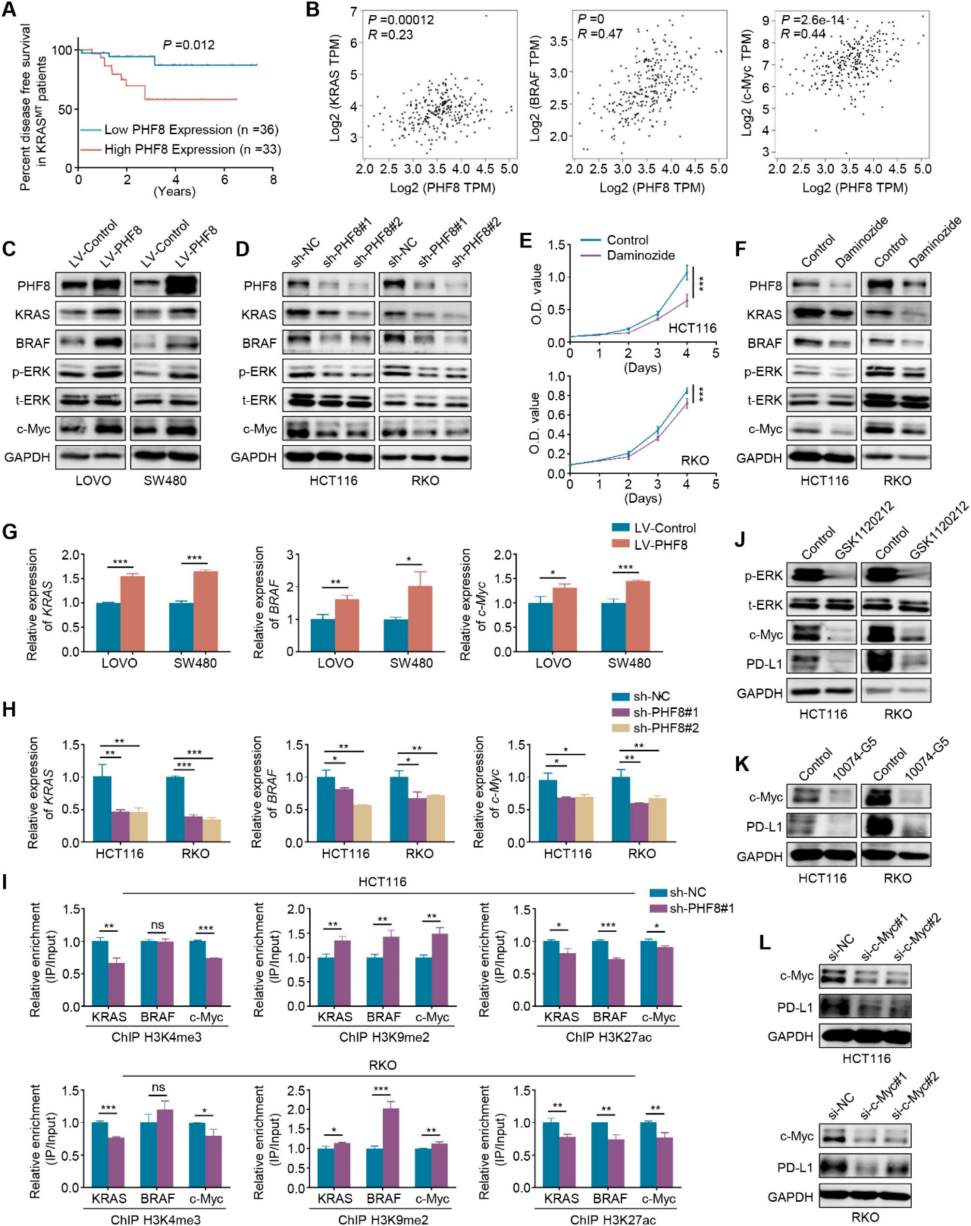
PHF8 remodels histone modifications within the promoters of *KRAS*, *BRAF*, and *c-Myc* to activate the MAPK/ERK/c-Myc signaling pathway and increase PD-L1 expression. **A**. The association of *PHF8* mRNA expression with disease-free survival in CRC patients with *KRAS* mutations. **B**. Correlation analysis of *PHF8* mRNA expression with mRNA levels of KRAS, BRAF, and c-Myc in CRCs using the GEPIA platform. **C**. Western blotting analysis was performed to evaluate the effect on ectopic expression of PHF8 in LOVO and SW480 cells on the expression of key genes in the MAPK/ERK pathway such as KRAS and BRAF as well as the levels of its downstream effectors such as phosphorylated ERK (p-ERK) and c-Myc. **D**. The effect of knockdown of PHF8 in HCT116 and RKO cells on the levels of the above genes was determined by western blotting analysis. **E**. HCT116 and RKO cells were treated with 50 µM PHF8 inhibitor daminozide for the indicated times, and its effect on cell proliferation was determined by MTT assays. **F**. HCT116 and RKO cells were treated with 50 µM daminozide for 48 h, and western blotting analysis was then used to evaluate its effect on the levels of KRAS, BRAF, p-ERK, and c-Myc. **G**, **H**. PHF8 was ectopically expressed in LOVO and SW480 cells (**G**) and PHF8 was knocked down in HCT116 and RKO cells (**H**), and their effect on the mRNA expression of KRAS, BRAF, and c-Myc was then assessed by qRT-PCR assays. *β-actin* was used as the normalized control. **I**. ChIP-qPCR assays were performed to detect the levels of H3K4me3, H3K27ac, and H3K9me2 within the promoters of KRAS, BRAF, and c-Myc in PHF8-knockdown HCT116 and RKO cells and control cells. **J**. HCT116 and RKO cells were treated with 0.5 µM MEK inhibitor GSK1120212 for 48 h, and its effect on the levels of p-ERK, c-Myc, and PD-L1 was then evaluated by western blotting analysis. **K**. HCT116 and RKO cells were treated with 50 µM c-Myc inhibitor 10,074-G5 for 48 h, and its effect on the expression of c-Myc and PD-L1 was then determined by western blotting analysis. **L**. c-Myc was knocked down in HCT116 and RKO cells, and western blotting analysis was then used to assess its effect on PD-L1 expression. GAPDH was used as a loading control for western blotting analysis. *P* value in A was determined by the log-rank test. *P* value in B was calculated by the log-rank test and the R-value was analyzed using Spearman’s correlation test. *P* value in E was calculated using One-way analysis of variance (ANOVA). *P* values in G-I were calculated using two-tailed unpaired Student’s t-tests. Data were presented as mean ± SD. *, *P* < 0.05; **, *P* < 0.01; ***, *P* < 0.001

*KRAS* and *BRAF* mutations, especially the former, are prevalent in CRC. These mutations promote the occurrence and progression of CRC by activating the MAPK/ERK signaling pathway and are strongly associated with poor patient prognosis [37–39]. By analyzing the TCGA database, we found that high expression of *PHF8* was significantly associated with a shorter disease-free survival time in KRAS-mutant CRC patients (Fig.  7A). Moreover, PHF8 was positively correlated with the expression levels of *KRAS*, *BRAF,* and *c-Myc (*Fig.  7B). These observations suggest that PHF8 may be involved in the activation of the MAPK/ERK pathway. As predicted, ectopic expression of PHF8 significantly increased the expression of KRAS, BRAF and c-Myc as well as the levels of phosphorylated ERK (p-ERK) in LOVO and SW480 cells (Fig.  7C), while knockdown of PHF8 in HCT116 and RKO cells displayed the opposite effect (Fig.  7D). Meanwhile, we treated HCT116 and RKO cells with PHF8 inhibitor daminozide and evaluated its effect on cell proliferation. The result indicated that daminozide significantly suppressed the proliferation of these two cancer cell lines (Fig.  7E). Similar to the results of PHF8 knockdown, daminozide dramatically decreased the levels of KRAS, BRAF, c-Myc, and p-ERK compared to the control (Fig.  7F).

We next assessed the effect of overexpression or knockdown of PHF8 on mRNA levels of *KRAS*, *BRAF* and *c-Myc*. The results expectedly demonstrated that ectopic expression of PHF8 elevated mRNA levels of these three genes (Fig. 7G), and vice versa (Fig. 7H). Mechanistically, PHF8 knockdown significantly decreased the levels of H3K4me3 or H3K27ac within the promoters of *KRAS*, *BRAF,* and *c-Myc*, while increasing the levels of H3K9me2 within their promoters (Fig. 7I). Considering that MAPK/ERK signaling pathway can upregulate the expression of PD-L1 [40, 41], we first treated HCT116 and RKO cells with MEK inhibitor GSK1120212 and found that the levels of c-Myc and PD-L1 were drastically reduced upon GSK1120212 treatment (Fig. 7J). Also, we treated HCT116 and RKO cells with c-Myc inhibitor 10,074-G5 or knocked down c-Myc in these two cell lines. The results showed that targeting c-Myc downregulated the expression of PD-L1 (Fig. 7K-L), which was consistent with the previous studies [42]. *More importantly*, *we assessed the effect of PHF8 overexpression or knockdown on the levels of key molecules in the MAPK/ERK/c-Myc signaling pathway and their downstream effector PD-L1* in vivo. *As expected*, *PHF8 overexpression significantly enhanced the levels of KRAS*, *BRAF*, *p-ERK*, *and c-Myc in the tumors*, and *vice versa* (Figure S17). The above findings, taken together, indicate that PHF8 activates the MAPK/ERK/c-Myc signaling pathway by remodeling histone modifications within the promoters of *KRAS*, *BRAF,* and *c-Myc*, thereby elevating the expression of PD-L1.

### c-Myc increases PHF8 expression by suppressing miR-22-3p

**Fig. 8:**
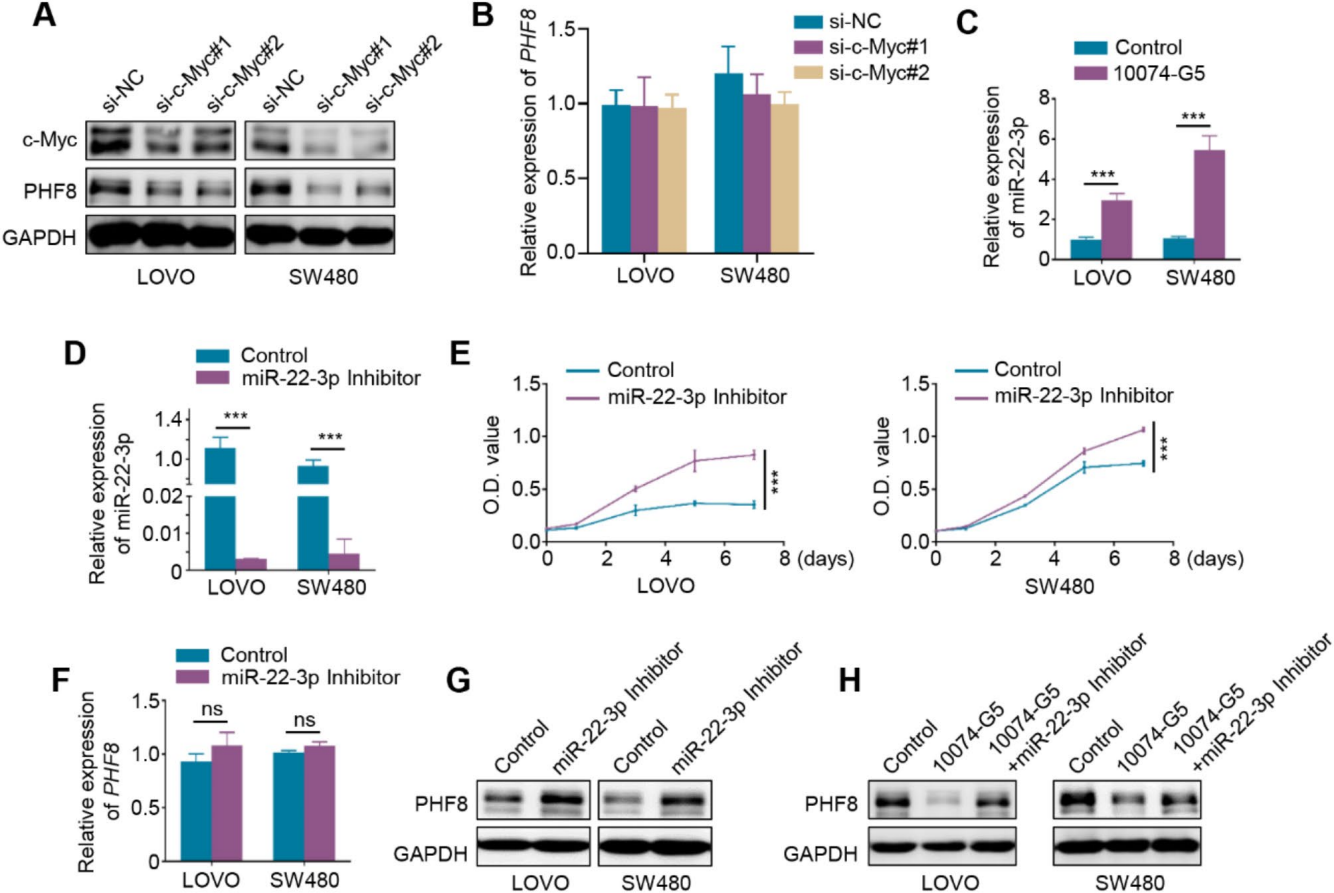
c-Myc upregulates PHF8 by inhibiting miR-22-3p. **A**, **B**. c-Myc was knocked down in LOVO and SW480 cells, and western blotting (**A**) and qRT-PCR (**B**) assays were used to determine its effect on protein and mRNA expression of PHF8. **C**. LOVO and SW480 cells were treated with 50 µM c-Myc inhibitor 10,074-G5 for 48 h, and its effect on miR-22-3p expression was then evaluated by qRT-PCR assay. **D-E**. LOVO and SW480 cells were transfected with miR-22-3p inhibitor (**D**), and its effect on cell proliferation was then determined by MTT assays (**E**). *U6* was used to normalize *miR-22-3p* expression. **F**, **G**. qRT-PCR (**F**) and western blotting (**G**) assays were used to evaluate the effect of miR-22-3p inhibitor on mRNA and protein expression of *PHF8* in LOVO and SW480 cells. *β-actin* was used as a normalized control for qRT-PCR assay, and GAPDH was used as a loading control for western blotting analysis. **H**. Western blotting analysis was performed to evaluate the effect of miR-22-3p inhibitor on PHF8 expression in LOVO and SW480 cells pretreated with 50 µM c-Myc inhibitor 10,074-G5. *P* value in E was calculated using One-way analysis of variance (ANOVA). *P* values in B-D and F were calculated using two-tailed unpaired Student’s t-tests. Data were presented as mean ± SD. ***, *P* < 0.001; ns, no significance

The c-Myc/miR-22-3p/PHF8 regulatory axis acts as a biological mechanism for the dysregulation of PHF8 expression in breast cancer and gastric cancer and plays an essential role in tumor progression [43, 44]. To confirm whether this regulatory mechanism is present in CRC, we knocked down c-Myc in LOVO and SW480 cells and demonstrated that c-Myc knockdown dramatically decreased the protein levels of PHF8 but not its mRNA levels (Fig. 8A-B). A similar result was also observed when these cell lines were treated with c-Myc inhibitor 10,074-G5 (Figure S18). These results suggest that c-Myc regulates PHF8 expression at the post-transcriptional levels. By analyzing the TCGA database, we found that there was a negative correlation between c-Myc and miR-22-3p in CRCs (Figure S19). As supported, miR-22-3p expression was significantly upregulated in LOVO and SW480 cells treated with c-Myc inhibitor 10,074-G5 compared to the control (Fig. 8C). Next, we transfected LOVO and SW480 cells with miR-22-3p inhibitor (Fig. 8D), and found that miR-22-3p inhibitor dramatically promoted the proliferation of LOVO and SW480 cells relative to the control (Fig. 8E). Also, we noted that miR-22-3p inhibitor had no effect on the mRNA levels of PHF8 but elevated its protein levels (Fig. 8F-G). In addition, we transfected miR-22-3p inhibitor into LOVO and SW480 cells that were pretreated with c-Myc inhibitor 10,074-G5. The results showed that the 10,074-G5 treatment dramatically downregulated PHF8 expression, while this effect could be partially reversed by a miR-22-3p inhibitor (Fig. 8H).

**Fig. 9:**
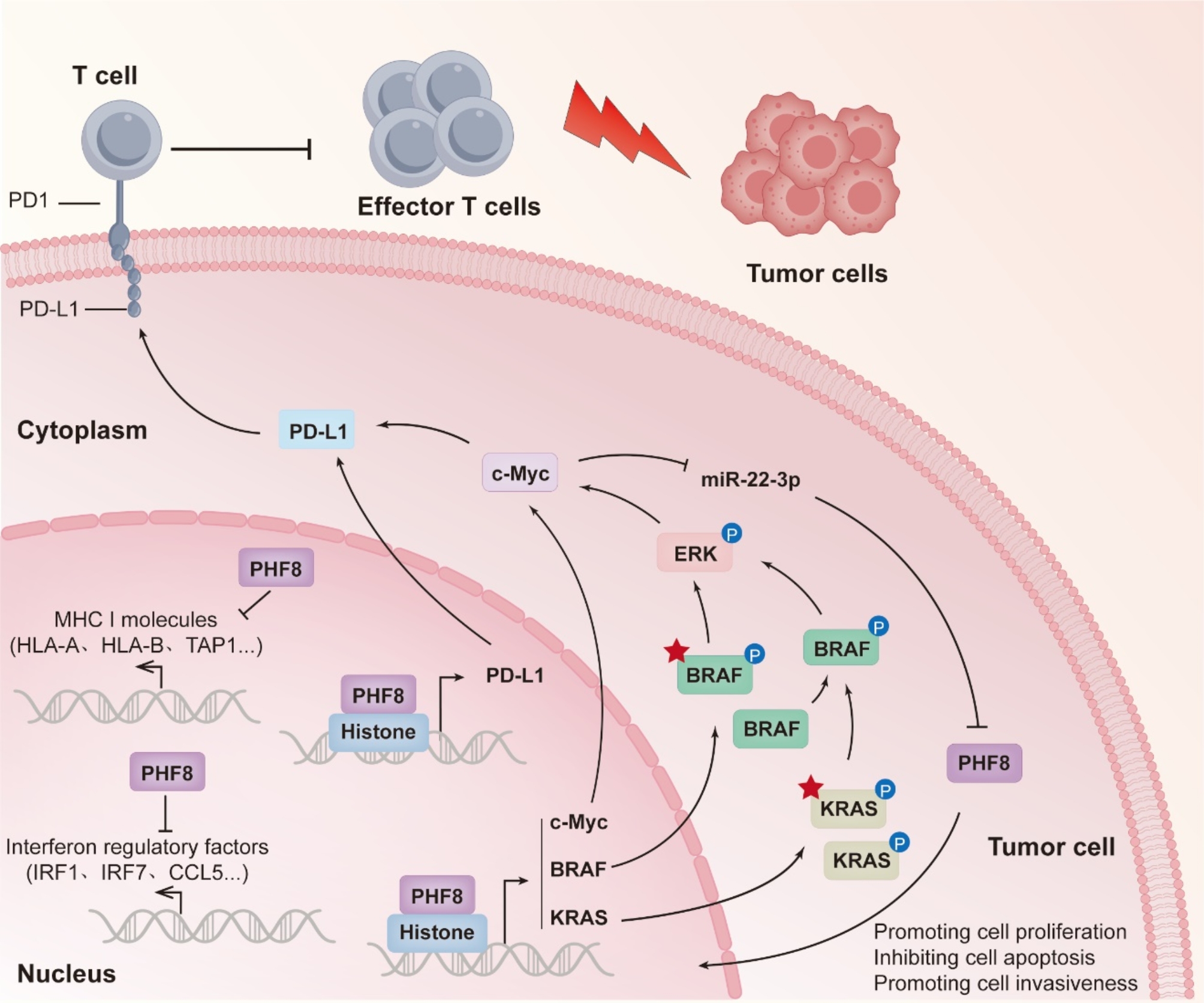
A schematic model of the mechanism by which PHF8 promotes malignant phenotypes and immune escape of KRAS- or BRAF-mutant CRC cells. Briefly, PHF8 downregulates the expression of MHC and interferon regulatory genes. On the other hand, PHF8 upregulates PD-L1 by remodeling histone modifications within its promoter, thereby leading to immune escape. Similarly, PHF8 also upregulates the expression of KRAS, BRAF, and c-Myc by remodeling histone modifications within their promoters to activate the MAPK/ERK/c-Myc signaling pathway. Thus, PHF8 may be a potential therapeutic target for KRAS- or BRAF-mutant CRCs. In addition, c-Myc can upregulate PHF8 at the post-transcriptional levels by downregulating miR-22-3p, while PHF8 in turn promotes c-Myc expression to form a positive feedback loop, ultimately promoting the malignant phenotypes and immune escape of KRAS- or BRAF-mutant CRC cells. Red asterisk represents mutant KRAS or BRAF.

Based on the above findings, we illustrate the molecular mechanism underlying specific oncogenic roles of PHF8 in KRAS- or BRAF-mutant CRCs (Fig. 9). In brief, PHF8 negatively regulates antigen presentation, interferon response, and chemokine expression. On the other hand, PHF8 promotes the transcription of PD-L1 by remodeling histone modifications within its promoter, thereby causing immune escape. In addition, PHF8 upregulates the expression of KRAS, BRAF, and c-Myc by reprogramming histone modifications within their promoters, thus activating the MAPK/ERK signaling pathway. As a result, PHF8 may serve as a potential therapeutic target for KRAS- or BRAF-mutant CRCs, while PHF8 further contributes to the immune escape of CRC cells via c-Myc-mediated PD-L1 upregulation. Also, c-Myc can increase the expression of PHF8 at the post-transcriptional levels by downregulating miR-22-3p. In turn, PHF8 promotes the expression of c-Myc to form a positive feedback loop, ultimately promoting the malignant phenotypes and immune escape of KRAS- or BRAF-mutant CRC cells.

## Discussion

CRC is a common tumor of the digestive tract, accounting for approximately 10% of all malignancies and ranking as the second leading cause of cancer-related death worldwide [45]. Due to the susceptibility to distant metastasis and the absence of viable treatment options, CRC patients’ chances of long-term survival are drastically lowered in most cases, and metastatic CRC remains incurable [46]. Mutations in the *KRAS* gene are an early event in the development of CRC and KRAS activation promotes cell proliferation, survival and metastasis by constitutively activating MAPK/ERK, PI3K/AKT, and NF-kB signaling pathways [11, 47]. Moreover, amplification or mutations of RTKs, RAS GTPase, and BRAF, for instance, activate their downstream signaling pathways and interact with inflammatory signaling pathways to control PD-L1 expression [40, 48].

The majority of *KRAS* mutations in CRC occur at codons 12, 13, and 61, of which the codon 12 mutation is the most prevalent among these mutations [49]. In addition, the G12D (glycine 12 to aspartic acid) and G12V (glycine 12 to valine) constitute the most common subtypes of CRC while G12C mutations account for only 7% of *KRAS* mutations (about 3% of CRC patients) [19]. For a long time in the past, the lack of surface-binding pockets and the strong affinity for GTP and GDP made it impossible to directly target RAS proteins with small molecules [19]. Encouragingly, the inhibitors targeting KRAS G12C mutations such as sotorasib (AMG-510) and adagrasib (MRTX849) have been approved for clinical use by the FDA in recent years and have achieved certain response results in NSCLC [50, 51]. However, these inhibitors targeting KRAS G12C mutations fail to achieve the ideal objective response in CRC patients, and the patients who respond early to inhibitors of KRAS G12C mutations quickly develop drug resistance [52]. The potential mechanisms involve secondary mutations in *KRAS* that hinder drug binding, and reactivation of upstream and downstream components in the MAPK/ERK signaling pathway [53]. Therefore, it is necessary to examine the potential advantages of combination targeting of critical cellular components to optimize the effectiveness of KRAS inhibition.

Considering the presence of oncogenic *KRAS* mutations leads to the overexpression of various cytokines and chemokines and inhibition of tumor-intrinsic interferon (IFN) signaling, thereby forming an immunosuppressive tumor microenvironment and ultimately promoting immune evasion of cancer cells [54, 55], combining immunotherapies may hold significant promise for the treatment of KRAS-mutant CRCs. Clinically, approximately 10–15% of CRC show *BRAF* mutations, with *BRAF*^*V600E*^ mutation being the most prevalent, causing the continuous activation of MAPK/ERK signaling pathway independently of RAS activity, which is suppressed by ERK-mediated negative feedback [15]. Currently, therapeutic drugs targeting mutations in *BRAF* have shown certain promising results; however, drug resistance has hindered clinical application, and challenges remain [23]. Thus, the identification and targeting of genes that are closely associated with KRAS or BRAF presents a promising therapeutic strategy for KRAS- and BRAF-mutated CRCs.

In the present study, we identified histone demethylase PHF8 as a negative regulator for the efficacy of anti-PD1 therapy using CRISPR-Cas9 gene knockout library. PHF8 has been reported to be overexpressed in different types of human cancers, including breast cancer [56], CRC [57], gastric cancer [58], prostate cancer [59] and hepatocellular carcinoma [60]. By bioinformatics analysis and experimental data obtained in this study, we found that the high expression of PHF8 was related to poor prognosis of CRC patients, inhibited antigen presentation and interferon response process, and antagonized the therapeutic effect of PD-1 antibody, which was consistent with a previous study showing that PHF8 could inhibit the intrinsic immune response [29]. These observations support the role of PHF8 as an immunosuppressive factor in CRC. Therefore, targeting PHF8 can specifically serve as a sensitization strategy for anti-PD1 therapy. In addition, we also explored the biological role of PHF8 in CRC and found that PHF8 played a pro-carcinogenic role in KRAS- or BRAF-mutant CRC cells, which was consistent with a previous study [34]. However, we did not observe similar results in MC38, CT26, and SW48 cells, which carry wild-type KRAS or BRAF. These observations, taken together, indicate that PHF8 may be a specific target for the treatment of KRAS- or BRAF-mutant CRCs.

PHF8 serves as a transcriptional co-activator by acting on monomethylated histone H4 lysine 20 (H4K20me1), monomethylated and dimethylated H3 lysine 9 (H3K9me1/2), and dimethylated H3 lysine 27 (H3K27me2) histone modifications [61]. In addition to the JmjC structural domain, PHF8 comprises a PHD structural domain that binds to the nucleosome of lysine trimethylated at position 4 (H3K4me3) at histone H3 [62]. H3K4me3 is predominantly located at the promoters of transcriptionally active genes and differentiation-related genes and is regarded as an active marker that binds to active transcriptional gene areas within chromatin [63, 64]. H3K27ac is localized to the promoter and enhancer regions of activated transcriptional genes and coexists with H3K4me3 [65, 66]. In addition, H3K27ac forms broader structural domains in intergenic areas, the so-called super-enhancers, to increase gene expression even further [67]. H3K9 methylation, particularly H3K9me2, and H3K9me3, is typically related to gene repression and heterochromatin formation [68]. In the present study, we proved that ectopic expression of PHF8 in KRAS- or BRAF-mutant CRC cells upregulated the expression of PD-L1, KRAS, BRAF, and c-Myc by increasing the levels of transcriptional activation marks H3K4me3 and H3K27ac and decreasing the levels of transcriptional repression mark H3K9me2 within their promoters. As a result, PFH8 led to the immune escape of CRC cells by elevating PD-L1 levels. On the other hand, PHF8 activated the MAPK/ERK signaling pathway by increasing the expression of KRAS and BRAF to promote the malignant progression of KRAS- or BRAF-mutant CRCs. Importantly, PHF8 could be in turn upregulated by the c-Myc/miR-22-3p signaling axis to form a positive feedback loop.

## Conclusion

The present study identifies PHF8 as an immunosuppressive molecule and demonstrates its oncogenic role in KRAS- or BRAF-mutant CRC. Specifically, PHF8 increases the transcriptional activities of PD-L1, KRAS, BRAF, and c-Myc to activate the MAPK/ERK pathway and upregulate PD-L1 by remodeling histone modifications within their promoters. In turn, PHF8 can also be elevated by c-Myc/miR-22-3p signaling axis to form a positive feedback loop, thereby promoting malignant phenotypes and immune escape of KRAS- or BRAF-mutant CRC cells. Thus, targeting PHF8 may be an effective therapeutic strategy for KRAS- or BRAF-mutant CRCs.

## Electronic supplementary material

Below is the link to the electronic supplementary material.

Supplementary Material 1

Supplementary Material 2

Supplementary Material 3

## Data Availability

The data in the study are available from the corresponding author on reasonable request.

## Abbreviations

ChIP: Chromatin immunoprecipitation
CRC: Colorectal cancer
HE: Hematoxylin-eosin
IHC: Immunohistochemistry
MHC: Major histocompatibility complex
OS: Overall Survival
PD-L1: Programmed death-ligand 1
PD1: Programmed cell death protein 1
PHF8: Plant homeodomain finger protein 8
RT-qPCR: Real-time quantitative polymerase chain reaction
TCGA: The Cancer Genome Atlas
WB: Western Blot
