# Supplementary material for: Genome-wide CRISPR screening identifies PHF8 as an effective therapeutic target for KRAS- or BRAF-mutant colorectal cancers
Source: J Exp Clin Cancer Res. 2025 Feb 25;44:70. doi: 10.1186/s13046-025-03338-2 (PMC11853609; doi:10.1186/s13046-025-03338-2)
Supplement: Supplementary file 1 — Supplementary Material 1 [file 13046_2025_3338_MOESM1_ESM.docx]

**Supplementary Data**

**Materials and Methods**

**Human datasets and data analysis**

*PHF8* mRNA expression in CRCs and non-cancerous colon tissues (control subjects) was obtained from the UCSC XENA database (<https://xenabrowser.net/datapages/>) [1]. The UALCAN database (http://ualcan.path.uab.edu/) was used to obtain the protein expression data of PHF8 in CRCs and control subjects, clinical staging data of CRC patients, and mRNA expression data of *PHF8* in patients with esophageal, gastric and rectal cancers [2]. Alterations in the *PHF8* gene, such as genomic amplification, mutations, and elevated mRNA expression, were obtained from the cBioPortal database (<http://www.cBioPortal.org/index.do>) [3]. *PHF8* mRNA expression and survival times for CRC patients were gathered from the OncoLnc data portal (<http://www.oncolnc.org>) [4]. The LinkedOmics (http://www.linkedomics.org) database was used to acquire the enrichment analysis of the *PHF8* gene set from 609 CRC patients [5]. The TISIDB database (http://cis.hku.hk/TISIDB) was used to analyze the associations of *PHF8* mRNA expression with tumor-infiltrating lymphocytes, MHC genes, and chemokines [6]. The TCGA dataset (https://portal.gdc.com) was used to download the mRNA expression dataset of *PHF8* and the related clinical data.

The correlations between gene expression and immunological score were plotted using the *R* software ggstatsplot package. All analysis methods and *R* packages were implemented by *R* v4.0.3 [7, 8]. Univariate and multivariate Cox regression analyses were used to choose the appropriate terms for the nomogram. Through the 'forest plot' R package, the forest was utilized to display the *P* value, HR, and 95% confidence interval (CI) of each variable. Based on the outcomes of the multivariate Cox proportional hazards analysis, a nomogram was created to forecast the overall recurrence over the next 5 years. The nomogram offered a graphical depiction of the elements that may be used to determine a patient's unique risk of recurrence based on the points assigned to each risk factor using the 'RMS' R program [9]. The TCGA dataset (https://portal.gdc.com) was used to retrieve the RNA-sequencing expression (level 3) profiles and the related clinical data for PHF8. The log-rank test was utilized to assess survival differences between the two groups. Using time ROC analysis, the prediction accuracy of *PHF8* mRNA was compared. The *P*-values and hazard ratio (HR) with a 95% CI for Kaplan-Meier curves were calculated using log-rank testing and univariate Cox proportional hazards regression [10, 11]. The GEPIA (http://gepia.cancer-pku.cn/) database was utilized to evaluate the correlations of *PHF8* mRNA expression with the mRNA expression of KRAS, BRAF, and c-Myc in CRC patients [12]. Using the StarBase platform (http://starbase.sysu.edu.cn/), we gathered correlation data between miR-22-3p and c-Myc expression in human CRC samples [13].

**References**

1. Goldman MJ, Craft B, Hastie M, Repečka K, McDade F, Kamath A et al. Visualizing and interpreting cancer genomics data via the Xena platform. Nat Biotechnol. 2020;38:675-678.
2. Chandrashekar DS, Karthikeyan SK, Korla PK, Patel H, Shovon AR, Athar M et al. UALCAN: An update to the integrated cancer data analysis platform. Neoplasia. 2022;25:18-27.
3. Cerami E, Gao J, Dogrusoz U, Gross BE, Sumer SO, Aksoy BA et al. The cBio cancer genomics portal: an open platform for exploring multidimensional cancer genomics data. Cancer Discov. 2012;2:401-404.
4. Anaya J. OncoLnc: linking TCGA survival data to mRNAs, miRNAs, and lncRNAs. PeerJ Computer Science. 2016;2.
5. Vasaikar SV, Straub P, Wang J, Zhang B. LinkedOmics: analyzing multi-omics data within and across 32 cancer types. Nucleic Acids Res. 2018;46:D956-d963.
6. Ru B, Wong CN, Tong Y, Zhong JY, Zhong SSW, Wu WC et al. TISIDB: an integrated repository portal for tumor-immune system interactions. Bioinformatics. 2019;35:4200-4202.
7. Iglesia MD, Parker JS, Hoadley KA, Serody JS, Perou CM, Vincent BG. Genomic Analysis of Immune Cell Infiltrates Across 11 Tumor Types. J Natl Cancer Inst. 2016;108.
8. Li T, Fu J, Zeng Z, Cohen D, Li J, Chen Q et al. TIMER2.0 for analysis of tumor-infiltrating immune cells. Nucleic Acids Res. 2020;48:W509-w514.
9. Jeong SH, Kim RB, Park SY, Park J, Jung EJ, Ju YT et al. Nomogram for predicting gastric cancer recurrence using biomarker gene expression. Eur J Surg Oncol. 2020;46:195-201.
10. Zhang Z, Lin E, Zhuang H, Xie L, Feng X, Liu J et al. Construction of a novel gene-based model for prognosis prediction of clear cell renal cell carcinoma. Cancer Cell Int. 2020;20:27.
11. Lin W, Wu S, Chen X, Ye Y, Weng Y, Pan Y et al. Characterization of Hypoxia Signature to Evaluate the Tumor Immune Microenvironment and Predict Prognosis in Glioma Groups. Front Oncol. 2020;10:796.
12. Tang Z, Li C, Kang B, Gao G, Li C, Zhang Z. GEPIA: a web server for cancer and normal gene expression profiling and interactive analyses. Nucleic Acids Res. 2017;45:W98-w102.
13. Li JH, Liu S, Zhou H, Qu LH, Yang JH. starBase v2.0: decoding miRNA-ceRNA, miRNA-ncRNA and protein-RNA interaction networks from large-scale CLIP-Seq data. Nucleic Acids Res. 2014;42:D92-97.

**Table S1**. Histopathological data of CRC patients

| Variables |  | Number | |
| --- | --- | --- | --- |
| Age |  |  |  |
| ≤60 |  |  | 4 |
| ＞60 |  |  | 18 |
| Gender |  |  |  |
| Female |  |  | 13 |
| Male |  |  | 9 |
| Tumor size |  |  |  |
| T1 |  |  | 0 |
| T2-T4 |  |  | 22 |
| Lymph node  metastasis |  |  |  |
| No |  |  | 9 |
| Yes |  |  | 13 |
| Distant metastasis |  |  |  |
| No |  |  | 19 |
| Yes |  |  | 3 |
| Clinical stage |  |  |  |
| I |  |  | 1 |
| II-IV |  |  | 21 |

**Table S2**. The antibodies used in this study

| Antibodies | Species | Catalog# | Source |
| --- | --- | --- | --- |
| Ki-67 | R | ab15580 | Abcam |
| PHF8 | R | ab36068 | Abcam |
| Anti-PD-L1 (Human) | R | GTX104763 | GeneTex |
| Anti-PD-L1 (IHC) | R | GB11339A | Servicebio |
| Anti-PD-L1 (Mouse) | R | ab213480 | Abcam |
| Anti-H3K4me3 | R | #9751 | Cell Signaling Technology |
| Anti-H3K9me2 | R | #4658 | Cell Signaling Technology |
| Anti-H3K27ac | R | #8173 | Cell Signaling Technology |
| Anti-Histone H3 | R | #14269 | Cell Signaling Technology |
| phospho-Erk1/2 (p-Erk1/2) | R | #4370 | Cell Signaling Technology |
| total Erk (t-Erk1/2) | R | #4695 | Cell Signaling Technology |
| GAPDH | R | AP0063 | Bioworld Technology |
| β-actin | R | #4970 | Cell Signaling Technology |
| N-cadherin | M | sc-8424 | Santa Cruz |
| MMP9 | M | sc-21733 | Santa Cruz |
| MMP2 | R | #40994 | Cell Signaling Technology |
| Slug | R | #9585 | Cell Signaling Technology |
| Snail1 | R | #3879 | Cell Signaling Technology |
| Vimentin | M | sc-6260 | Santa Cruz |
| Anti-Histone H3 | R | #14269 | Cell Signaling Technology |
| Anti-mouse CD3 | R | 100235 | Biolegend |
| Anti-mouse CD4 | R | 100405 | Biolegend |
| Anti-mouse CD8 | R | 100705 | Biolegend |
| Anti-mouse CD45 | R | 103105 | Biolegend |
| Anti-KRAS | R | 12063-1-AP | Proteintech |
| Anti-BRAF | M | sc-5284 | Santa Cruz |
| Anti-c-Myc | R | ab32072 | Abcam |

R: rabbit; M: mouse

**Table S3**. The siRNA sequences used in this study

| **siRNAs** | **Sequence (5’-3’)** |
| --- | --- |
| si-Phf8#1 (Mouse) | CGGTGGAATTCTTGATCTA |
| si-Phf8#2(Mouse) | GAAGGTAGACAGTGCAAGA |
| si-PHF8#1 (Human) | GCTGGCCAGTTGAGCTATAAT |
| si-PHF8#2 (Human) | CCCAACTGTGAAGTCTTGCAT |

**Table S4**. The qRT-PCR primers used in this study

| **Genes** | **Forward primer (5’-3’)** | **Reverse primer (5’-3’)** |
| --- | --- | --- |
| *β-actin (H)* | CACCATTGGCAATGAGCGGTTC | AGGTCTTTGCGGATGTCCACGT |
| *PHF8 (H)* | GGACACATACAGTCATCAGGCAC | GGCTCTCATTTCCATCAAGGTCC |
| *N-cadherin (H)* | CCTCCAGAGTTTACTGCCATGAC | GTAGGATCTCCGCCACTGATTC |
| *Slug (H)* | ATCTGCGGCAAGGCGTTTTCCA | GAGCCCTCAGATTTGACCTGTC |
| *Snail (H)* | TGCCCTCAAGATGCACATCCGA | GGGACAGGAGAAGGGCTTCTC |
| *Vimentin (H)* | AGGCAAAGCAGGAGTCCACTGA | ATCTGGCGTTCCAGGGACTCAT |
| *MMP2 (H)* | AGCGAGTGGATGCCGCCTTTAA | CATTCCAGGCATCTGCGATGAG |
| *MMP9 (H)* | GCCACTACTGTGCCTTTGAGTC | CCCTCAGAGAATCGCCAGTACT |
| *HLA-A (H)* | ACCCTCGTCCTGCTACTCTC | CTGTCTCCTCGTCCCAATACT |
| *HLA-B (H)* | CAGTTCGTGAGGTTCGACAG | CAGCCGTACATGCTCTGGA |
| *HLA-C (H)* | GGACAAGAGCAGAGATACACG | CAAGGACAGCTAGGACAACC |
| *HLA-H (H)* | GTCTGGCACCCTAGTCATTG | ACGTTCAGCTAAGACGTAGTGC |
| *TAP1(H)* | CTGGGGAAGTCACCCTACC | CAGAGGCTCCCGAGTTTGTG |
| *TAP2 (H)* | TGGACGCGGCTTTACTGTG | GCAGCCCTCTTAGCTTTAGCA |
| *ERAP1 (H)* | CCCCTCAAATGGTCCCTTGC | GAGATGCTTCAGTGCTCTGAC |
| *ERAP2 (H)* | CACTAATGGGGAACGATTTCCTT | CTGACCAAGACTTCGATCTTCTC |
| *B2M (H)* | GAGGCTATCCAGCGTACTCCA | CGGCAGGCATACTCATCTTTT |
| *PSMB8 (H)* | TCTCCAGAGCTCGCTTTACC | CACTCCATGCTGGAACTTGA |
| *PSMB9 (H)* | CGTTGTGATGGGTTCTGATTCC | GACAGCTTGTCAAACACTCGGTT |
| *IRF1 (H)* | AACCAAATCCCGGGGCTCAT | TGTTGATGTCCCAGCCATGC |
| *IRF7 (H)* | CCGGGAGCTGTGCTGG | GGAGTCCAGCATGTGTGTGT |
| *CCL5 (H)* | AGCAGTCGTCCACAGGT | CGGTTCTTTCGGGTGACAAA |
| *CXCL10 (H)* | TGCCATTCTGATTTGCTGCC | ATGCTGATGCAGGTACAGCG |
| *STAT1 (H)* | ATGCTGGCACCAGAACGAAT | CAGAGAGGTCGTCTCGAGGT |
| *PD-L1 (H)* | TGCCGACTACAAGCGAATTACTG | CTGCTTGTCCAGATGACTTCGG |
| *β-actin (M)* | CATTGCTGACAGGATGCAGAAGG | TGCTGGAAGGTGGACAGTGAGG |
| *PHF8 (M)* | GGACCTTGATGGCAGTGAGAAC | CCTGTTCTGCTACCAGTCTTGC |
| *H2-K1 (M)* | GCTGGTGAAGCAGAGAGACTCAG | GGTGACTTTATCTTCAGGTCTGCT |
| *H2-D1 (M)* | AGTGGTGCTGCAGAGCATTACAA | GGTGACTTCACCTTTAGATCTGGG |
| *Tap1 (M)* | AGTCTGGAGCCCACGATTTCATC | GGGTGATAAGAAGAACCGTCCG |
| *Tapbp (M)* | GGCCTGTCTAAGAAACCTGCC | CCACCTTGAAGTATAGCTTTGGG |
| *Erap1 (M)* | TAATGGAGACTCATTCCCTTGGA | AAAGTCAGAGTGCTGAGGTTTG |
| *B2m (M)* | TGGTGCTTGTCTCACTGACC | TTCAGTATGTTCGGCTTCCC |
| *Psmb6 (M)* | GGACAACCACTGGGTCCTAC | CAAGCTGGTAAGTGACAGCGT |
| *Psmb7 (M)* | GTGTCGGTGTTTCAGCCAC | GTGCCAGTTTTCCGAGCTTTC |
| *Psmb8 (M)* | GTGCAGGTTGTATTATCTTCGGA | CGAGTCCCATTGTCATCTACG |
| *Psmb10 (M)* | GAGGAATGCGTCCTTGGAACA | CACAACCGAATCGTTAGTGGC |
| *IRF1 (M)* | TCCAAGTCCAGCCGAGACACTA | ACTGCTGTGGTCATCAGGTAGG |
| *IRF7 (M)* | AAGCTGGAGCCATGGGTATG | GACCCAGGTCCATGAGGAAG |
| *IRF9 (M)* | ACAACTGAGGCCACCATTAGAGA | CACCACTCGGCCACCATAG |
| *CCL5 (M)* | CCCTCACCATCATCCTCACT | CCTTCGAGTGACAAACACGA |
| *CXCL9 (M)* | CCTAGTGATAAGGAATGCACGATG | CTAGGCAGGTTTGATCTCCGTTC |
| *CXCL10 (M)* | AGGGGAGTGATGGAGAGAGG | TGAAAGCGTTTAGCCAAAAAAGG |
| *CXCL11 (M)* | CCGAGTAACGGCTGCGACAAAG | CCTGCATTATGAGGCGAGCTTG |
| *STAT1 (M)* | TATTCCAGACCAAAGGAAGCAC | GAAGGGTGGACTTCAGACACAG |
| *PD-L1 (M)* | TGCGGACTACAAGCGAATCACG | CTCAGCTTCTGGATAACCCTCG |
| *KRAS (H)* | CAGTAGACACAAAACAGGCTCAG | TGTCGGATCTCCCTCACCAATG |
| *BRAF (H)* | AACGAGACCGATCCTCATCAGC | GGTAGCAGACAAACCTGTGGTTG |
| *c-Myc (H)* | CCTGGTGCTCCATGAGGAGAC | CAGACTCTGACCTTTTGCCAGG |
| *U6 (H)* | GCTTCGGCAGCACATATACTAAAAT | CGCTTCACGAATTTGCGTGTCAT |
| *miR-22-3p (H)* | TGCTAAGCTGCCAGTTGAA | ATCCAGTGCGTGTCGTG |

H: human; M: mouse

**Table S5**. The primer sequences for ChIP-qPCR analysis

| **DNA fragment** | **Forward primer (5’-3’)** | **Reverse primer (5’-3’)** |
| --- | --- | --- |
| PD-L1 | AGGTAGGGAGCGTTGTTCCT | CCCTTCTCCTCTCTCCATCC |
| PD-L1 | GATGCTAGGCTGGAGGTCTG | ATGCTGCAGCTGGGATAACT |
| KRAS | CAGGTGCGGGAGAGAGGTA | GGGGACCCCTAATTCATTCA |
| BRAF | TTCATCAAGCAGCCTCACAC | TCACCAGCGACCTCCTTACT |
| c-Myc | GGAAAACGGGAATGGTTTTT | TATGCGGTCCCTACTCCAAG |


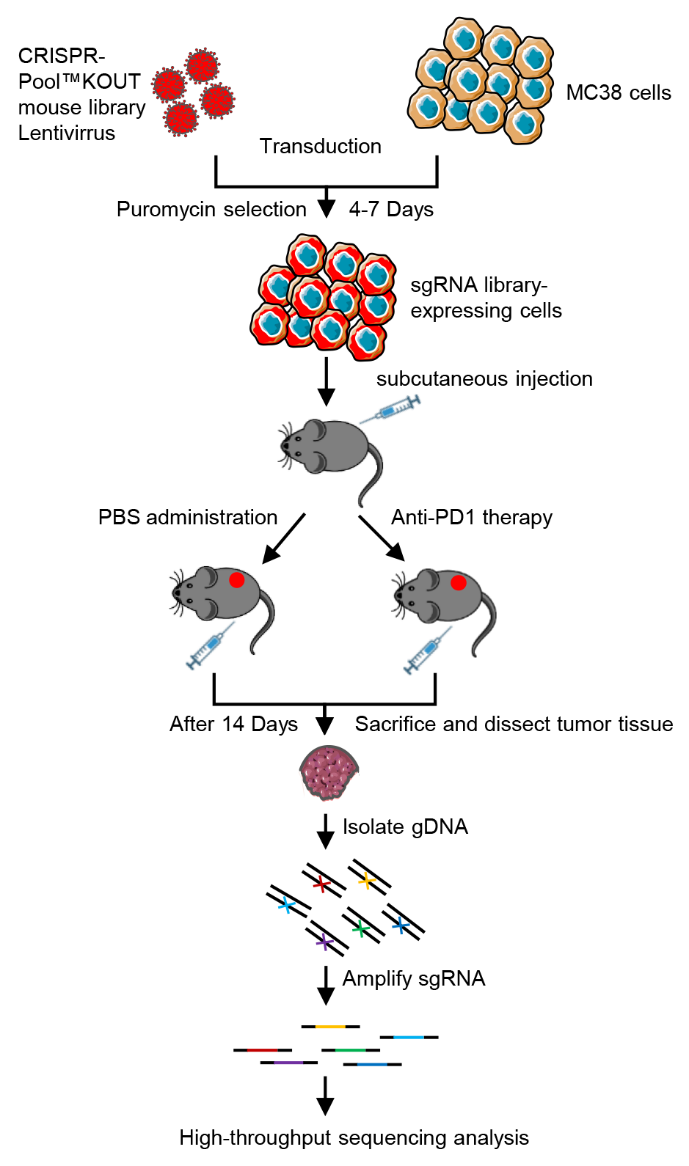


**Figure S1**. Schematic diagram of genome-scale Cas9 knockout screening.


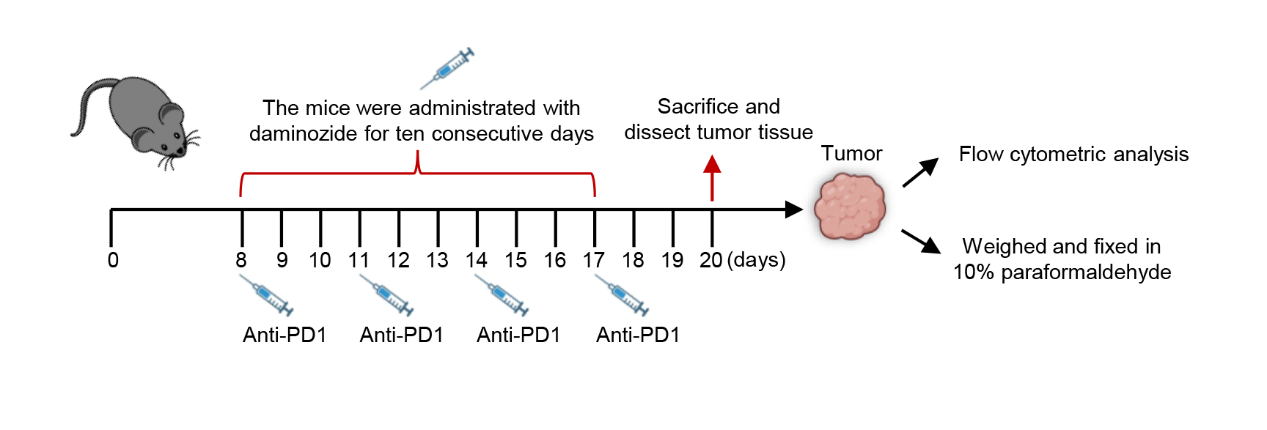


**Figure S2.** Schematic diagram for mice with different drug treatment strategies.


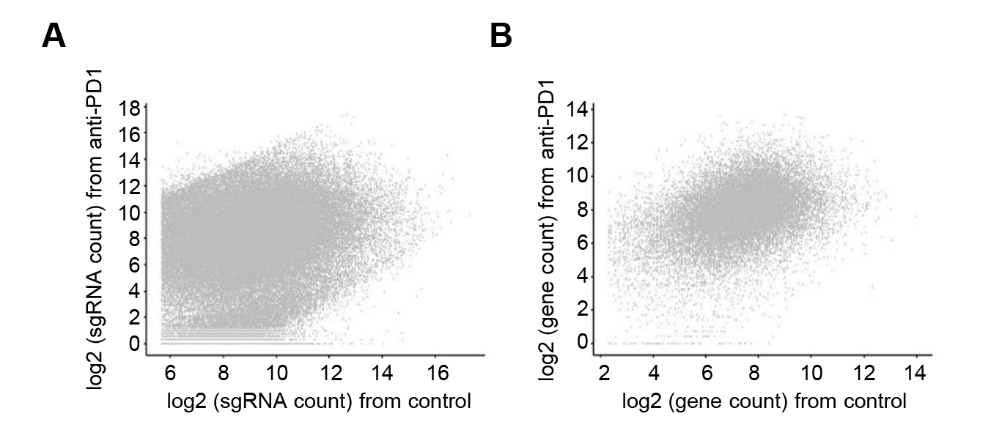


**Figure S3.** Sequencing and gene distribution of sgRNAs. **A**. sgRNAs and gene enumeration. **B**. Overall distribution of sgRNAs.


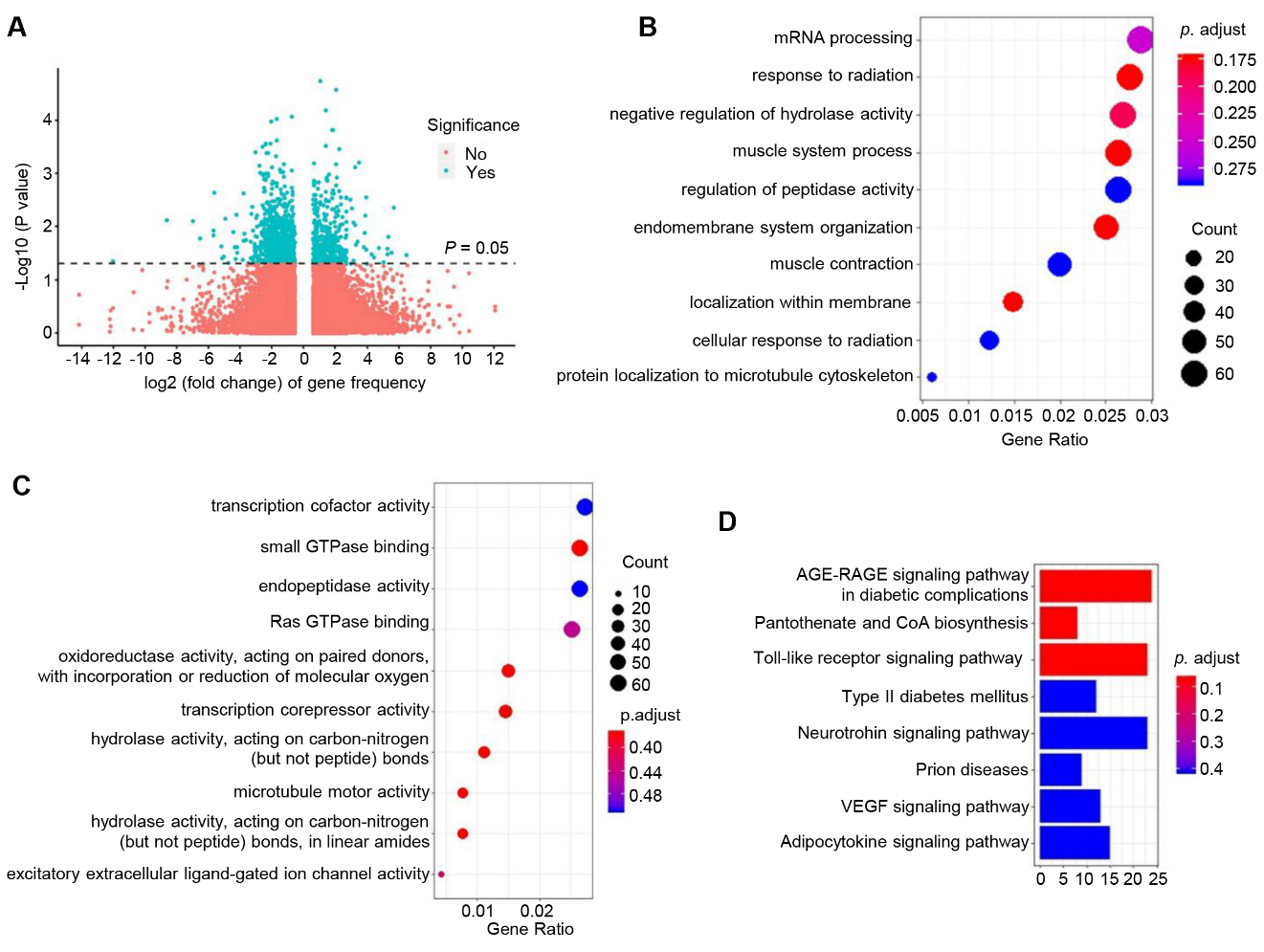


**Figure S4.** GO and KEGG analysis of differential genes. **A**. Distribution of different frequency of gene representation in the PD1 antibody-treated group compared with the control by the MAGeCK software analysis. **B, C**. GO analysis of biological processes (B) and molecular function (C) of differential genes. **D**. KEGG pathway analysis of deleted genes.

**
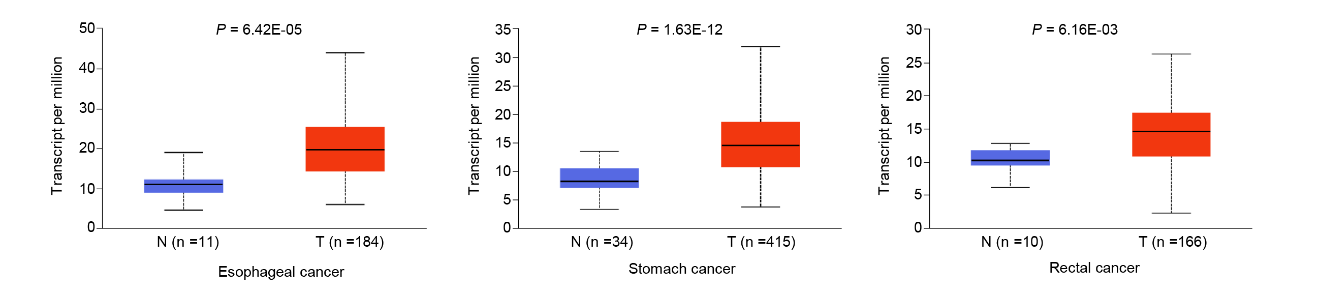
**

**Figure S5.** mRNA expression of *PHF8* in digestive tract tumors. *PHF8* mRNA expression in esophageal cancer, stomach cancer, and rectal cancer (data from UALCAN platform). *P* values was calculated using two-tailed unpaired Student’s t-tests. T, tumor tissue; N, normal control.


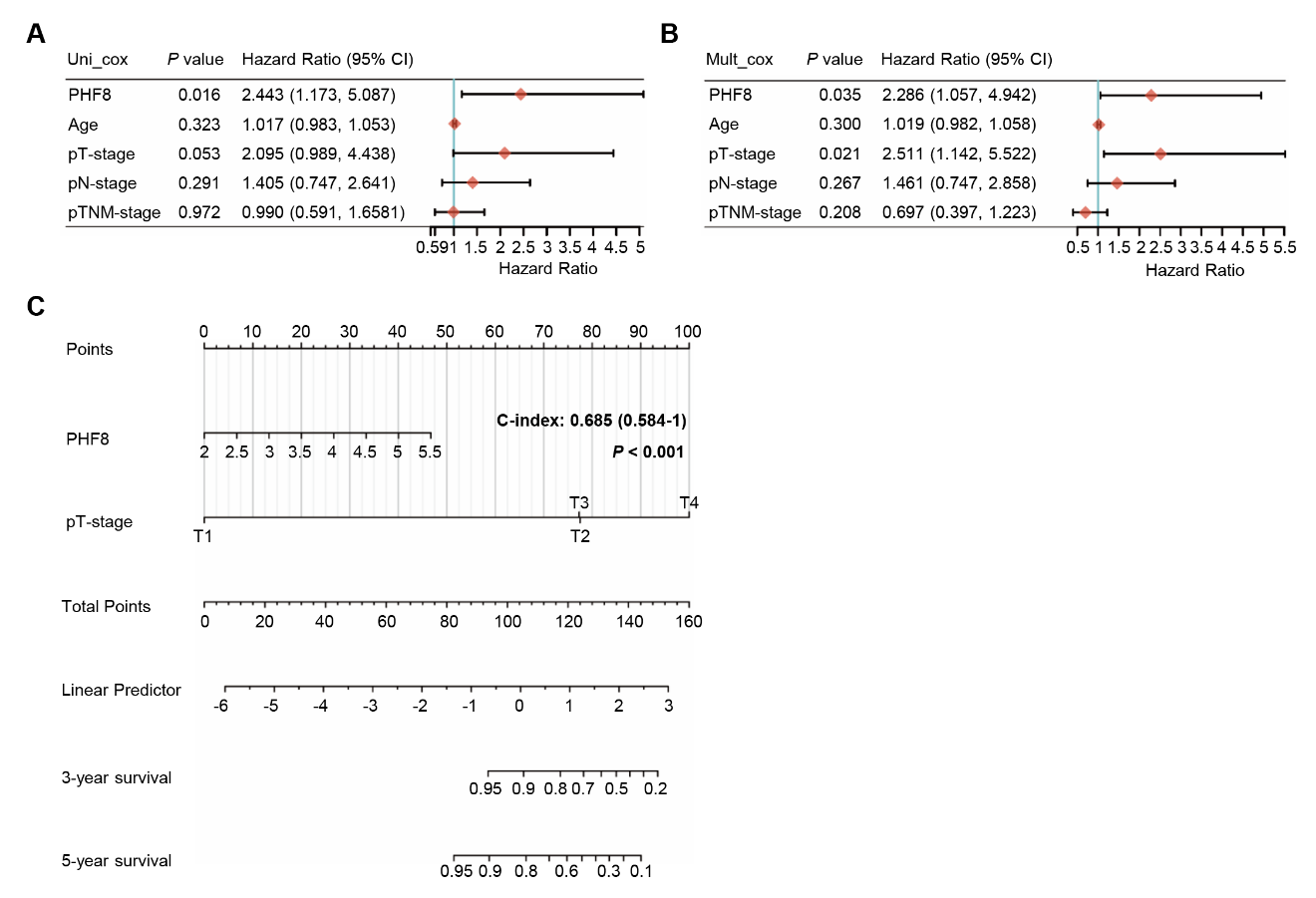


**Figure S6.** Correlation between *PHF8* mRNA expression and prognosis of CRC patients. **A**. Univariate prognostic analysis of CRC patients, including *PHF*8 mRNA expression, age, T stage, lymph node N stage, and clinical TNM stage. **B**. Multivariate prognostic analysis of CRC patients. **C**. Nomographic prognostic analysis of CRC patients, in which a line is drawn vertically from the corresponding axis of each risk factor until it reaches the top line labeled "Points." Summarize the number of points for all risk factors and then draw a line from the axis labeled "Total Points" until it intercepts each survival axis to determine the probability of survival at 3 and 5 years. *P*-values and hazard ratio (HR) with a 95% CI for Kaplan-Meier curves were calculated using log-rank testing and univariate Cox proportional hazards regression.


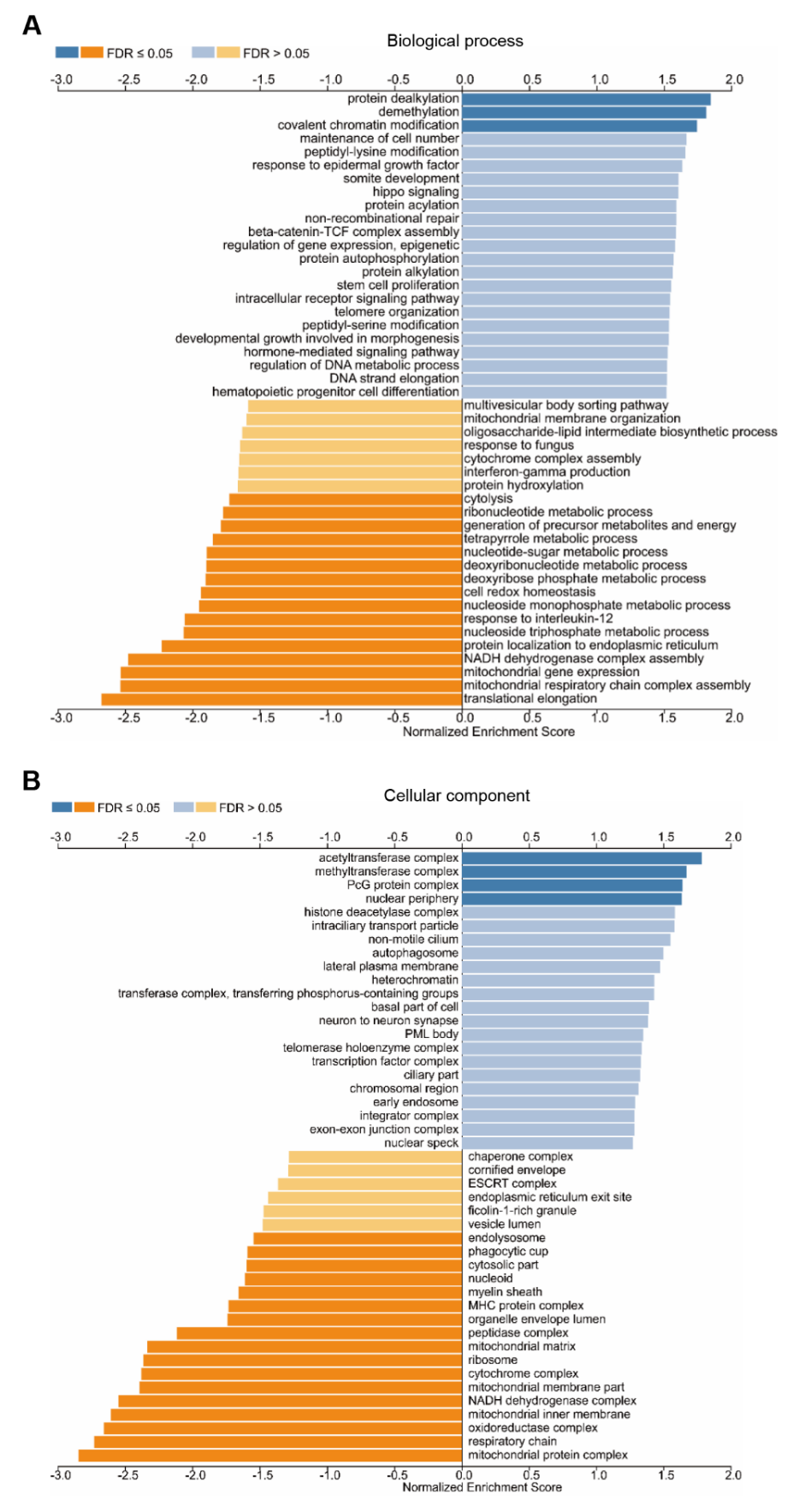


**Figure S7.** Gene set enrichment analysis of PHF8 in CRCs. **A**. Biological process regulated by PHF8. **B**. Cellular component modulated by PHF8.

**
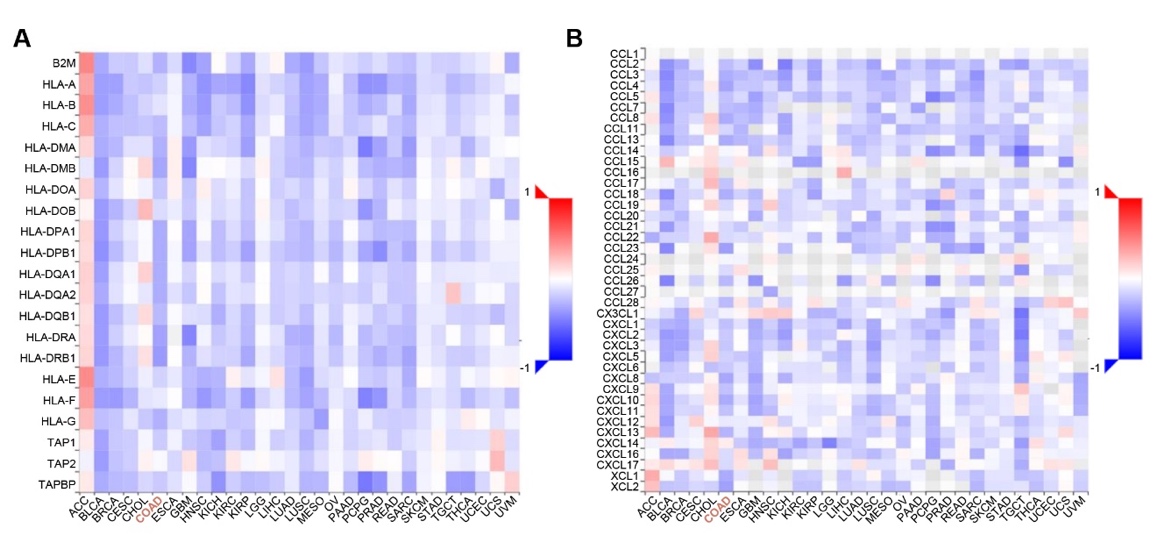
**

**Figure S8. A**. Correlation of *PHF8* mRNA expression with the expression of MHC genes and (B) chemokines.


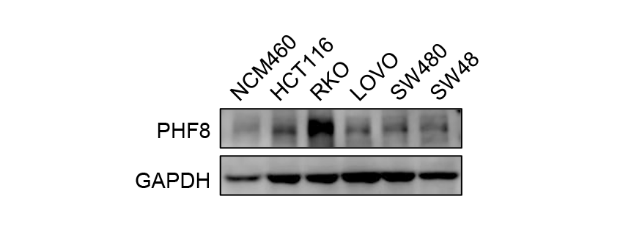


**Figure S9.** Basal expression of PHF8 in human normal colonic epithelial cell line and CRC cell lines.


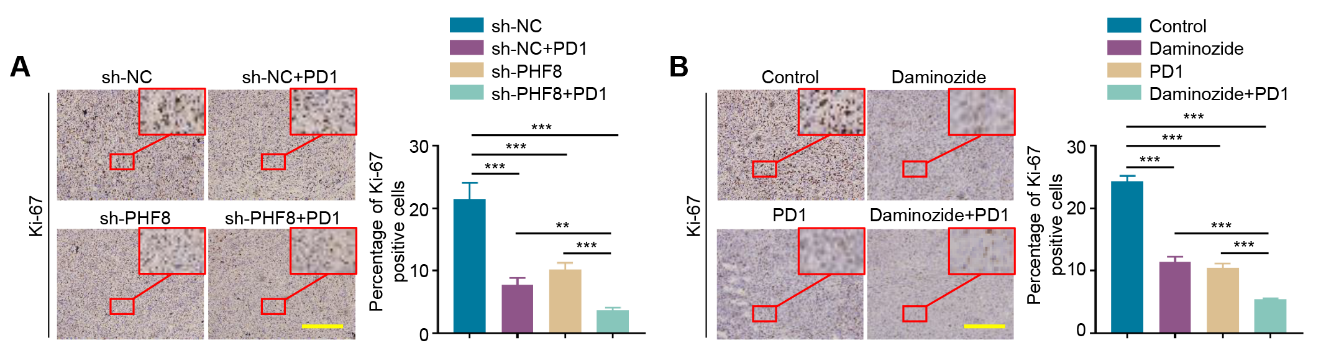


**Figure S10.** PHF8 inhibition improves anti-tumor efficacy of PD1 antibody. **A**. Left panel shows representative IHC staining of Ki-67 in the indicated xenograft tumors (including sh-NC, sh-NC+PD1 antibody, sh-PHF8, and sh-PHF8+PD1 antibody), and the right panel shows the statistical analysis on the percentage of Ki-67-positive cells. **B**. Representative IHC staining of Ki-67 in the indicated xenograft tumors (including control, PD1 antibody, PHF8 inhibitor daminozide, and daminozide+PD1 antibody) was shown in the left panel. Statistical analysis on the percentage of Ki-67-positive cells was shown in the right panel. Scale bars, 200 µm. *P* values were calculated using two-tailed unpaired Student’s t-tests. Data were presented as mean ± SD. **, *P* < 0.01; ***, *P* < 0.001.


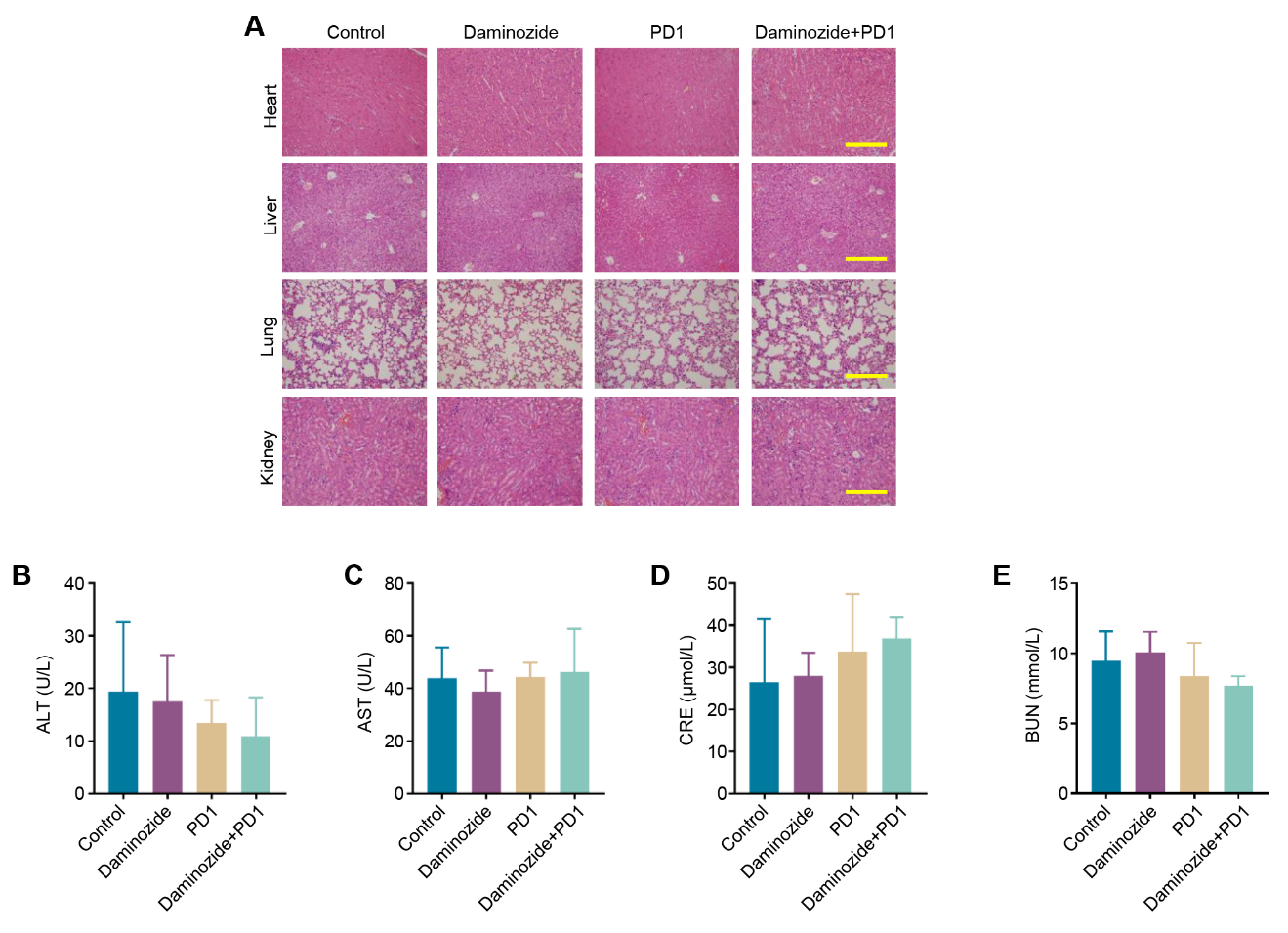


**Figure S11.** *In vivo* safety evaluation of PHF8 inhibitor daminozide combined with PD1 antibody. **A**. Representative H&E staining of heart, liver, lung, and kidney tissues in mice with different treatments including control, PD1 antibody, PHF8 inhibitor daminozide, and daminozide in combination with PD1 antibody. Scale bars, 200 µm. **B-E**. ELISA assays were performed to evaluate the effect of the above treatments on the levels of ALT (B), AST (C), CRE (D), and BUN (E). *P* values were calculated using two-tailed unpaired Student’s t-tests. Data were presented as mean ± SD.


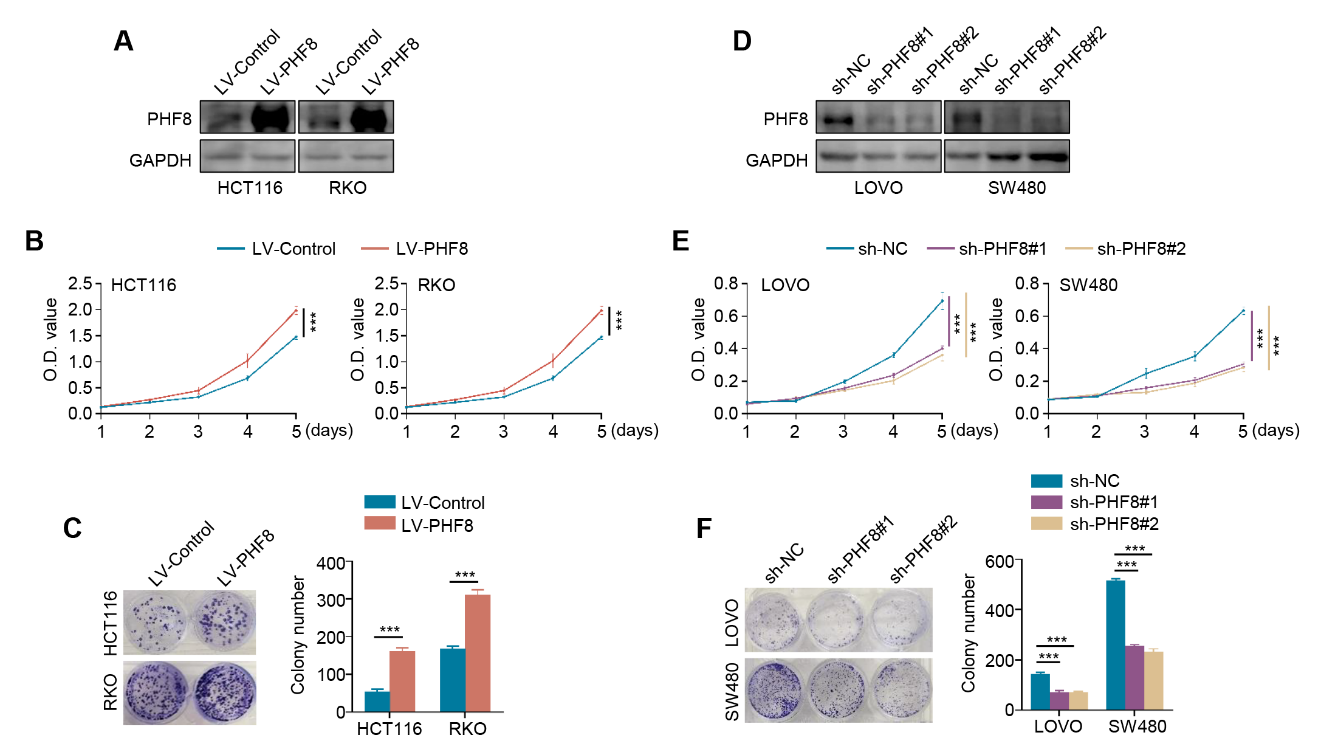


**Figure S12.** PHF8 promotes the proliferation and colony formation of BRAF- or KRAS-mutant CRC cells. **A**. Ectopic expression of PHF8 in HCT116 and RKO cells was confirmed by western blotting analysis. GAPDH was used as a loading control. **B**. MTT assay showing the effect of PHF8 overexpression on the proliferation of HCT116 and RKO cells. **C**. The effect of PHF8 overexpression on colony formation ability of HCT116 and RKO cells. The left panel shows representative images of colony formation, and the right panel shows a quantitative analysis of colony numbers. **D**. Knockdown of PHF8 in LOVO and SW480 cells was validated by western blotting analysis. GAPDH was used as a loading control. **E**. MTT assays were performed to assess the effect of PHF8 knockdown on the proliferation of LOVO and SW480 cells. **F**. The effect of PHF8 knockdown on colony formation ability of LOVO and SW480 cells. Representative images of colony formation were shown in the left panel, and quantitative analysis of colony numbers was shown in the right panel. *P* values in B and E were calculated using One-way analysis of variance (ANOVA). *P* values in C and F were calculated using two-tailed unpaired Student’s t-tests. Data were presented as mean ± SD. *, *P* < 0.05; **, *P* < 0.01; ***, *P* < 0.001.


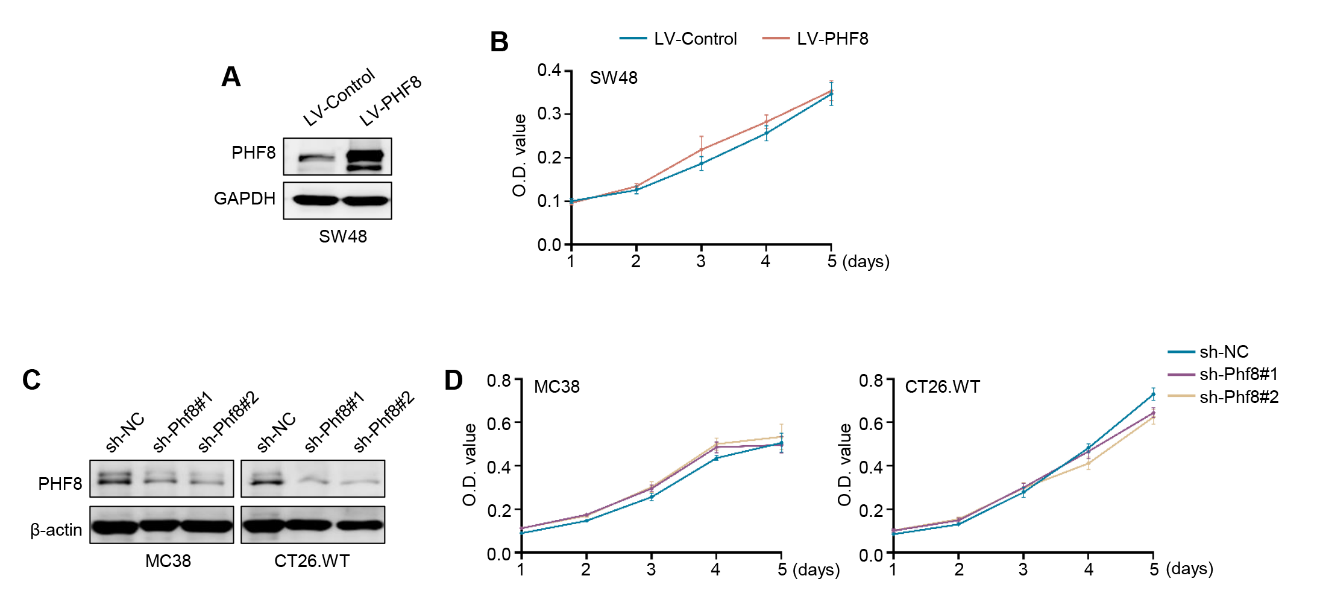


**Figure S13.** The effect of PHF8 on the proliferation of KRAS wild-type CRC cells. **A**. Ectopic expression of PHF8 in SW48 cells was confirmed by western blotting analysis. GAPDH was used as a loading control. **B**. The effect of PHF8 overexpression on the proliferation of SW48 cells by MTT assay. **C**. Knockdown of PHF8 in MC38 and CT26.WT cells were confirmed by western blotting analysis. β-actin was used as a loading control. **D**. The effect of PHF8 knockdown on the proliferation of MC38 and CT26.WT cells by MTT assay. *P* values were calculated using One-way analysis of variance (ANOVA). Data were presented as mean ± SD.


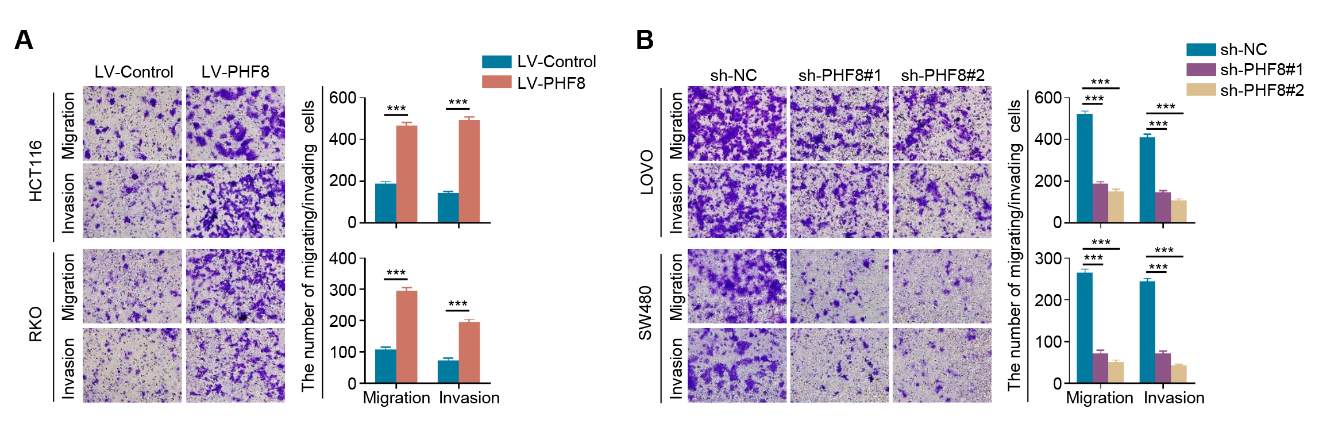


**Figure S14.** The effects of PHF8 overexpression in HCT116 and RKO cells (**A**) and PHF8 knockdown in LOVO and SW480 cells (**B**) on cell migration and invasion potential . Representative images of migrated/invaded cells were shown in the left panel, and statistical analysis on the number of migrated/invaded cells was shown in the right panel. *P* values were calculated using two-tailed unpaired Student’s t-tests. Data were presented as mean ± SD. ***, *P* < 0.001.


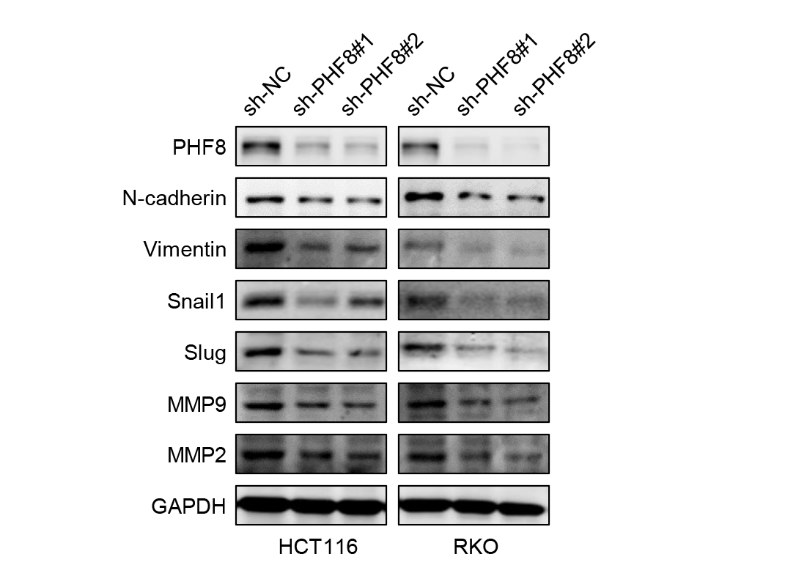


**Figure S15.** The effect of PHF8 on the expression of metastasis-related genes in CRC cells. PHF8 was knocked down in HCT116 and RKO cells, and western blotting analysis was then performed to assess its effect on the expression of metastasis-related genes, including N-cadherin, Vimentin, Snail1, Slug, MMP9, and MMP2. GAPDH was used as a loading control.


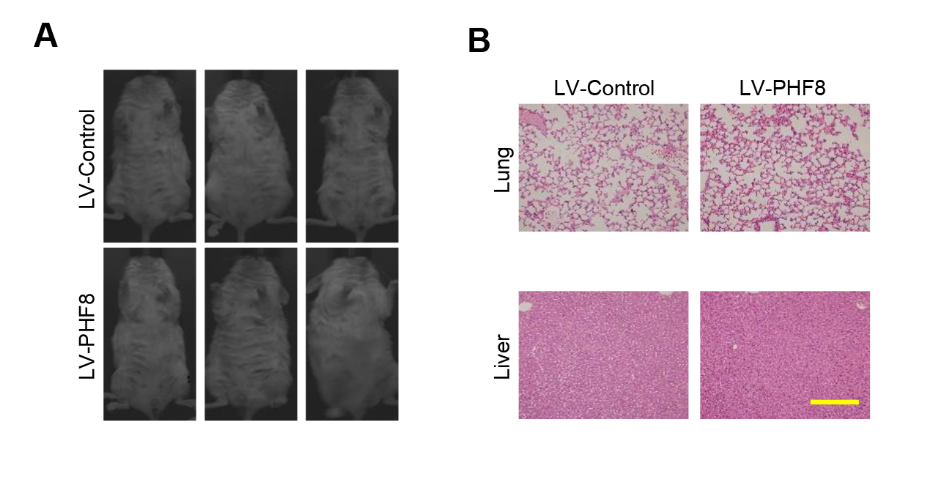


**Figure S16.** The effect of PHF8 on the metastasis ability of KRAS or BRAF wild-type CRC cells. **A**. The effect of tail vein injection of PHF8-overexpression SW48 cells and control cells was evaluated by an *in vivo* imaging system. **B**. Representative H&E images of lung and liver tissues of mice after tail vein injection of PHF8-overexpression SW48 cells and control cells. Scale bars, 200 µm.


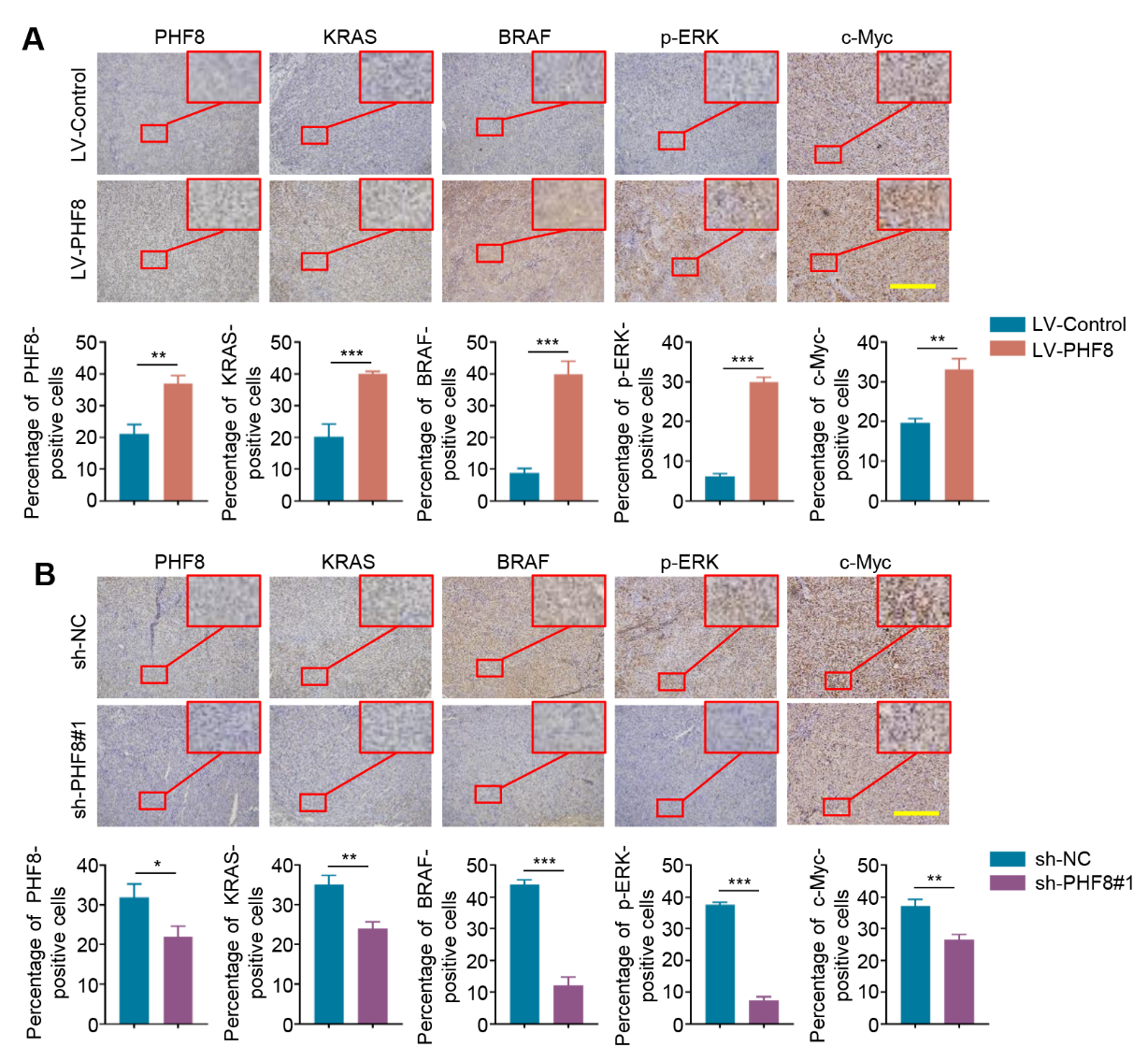


**Figure S17.** The effect of PHF8 overexpression (**A**) or knockdown (**B**) on the levels of key molecules in the MAPK/ERK/c-Myc signaling pathway and their downstream effector PD-L1. Upper panels show the representative IHC staining of PHF8, KRAS, BRAF, pERK, and c-Myc in the indicated tumors, and lower panels show statistical analysis on the percentage of positive cells. Scale bars, 200 µm. *P* values were calculated using two-tailed unpaired Student’s t-tests. Data were presented as mean ± SD. *, *P* < 0.05; **, *P* < 0.01; ***, *P* < 0.001.

**
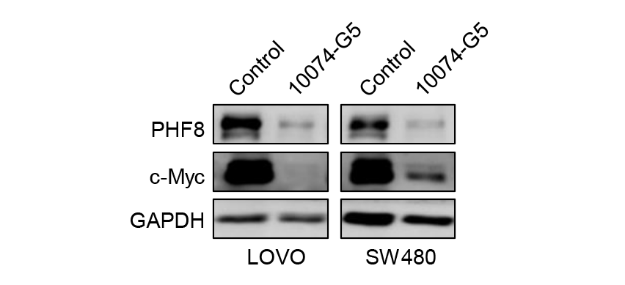
**

**Figure S18.** The effect of c-Myc on PHF8 expression in CRC cells. LOVO and SW480 cells were treated with 50 μM c-Myc inhibitor 10074-G5 for 48 h, and its effects on PHF8 expression were then evaluated by western blotting analysis. GAPDH was used as a loading control.


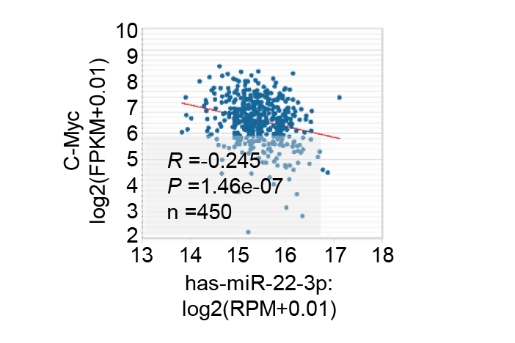


**Figure S19.** Correlation analysis between the expression of c-Myc and miR-22-3p in CRCs using the StarBase database. *P* values was calculated by the log-rank test and the R-value was analyzed using Spearman’s correlation test.
